# Sputum Microbiota Compositions Correlate With Metabolome and Clinical Outcomes of COPD‐Bronchiectasis Association: A Prospective Cohort Study

**DOI:** 10.1002/EXP.20240149

**Published:** 2025-05-04

**Authors:** Zhen‐feng He, Xiao‐xian Zhang, Cui‐xia Pan, Xin‐zhu Yi, Yan Huang, Chun‐lan Chen, Shan‐shan Zha, Lai‐jian Cen, Han‐qin Cai, Lei Yang, Jia‐qi Gao, Hui‐min Li, Zhen‐hong Lin, Sheng‐zhu Lin, Zhang Wang, Nan‐shan Zhong, Wei‐jie Guan

**Affiliations:** ^1^ Department of Allergy and Clinical Immunology Department of Respiratory and Critical Care Medicine State Key Laboratory of Respiratory Diseases National Clinical Research Center for Respiratory Disease National Center for Respiratory Medicine Guangzhou Institute of Respiratory Health First Affiliated Hospital Guangzhou Medical University Guangzhou Guangdong P.R. China; ^2^ Institute of Ecological Sciences School of Life Sciences South China Normal University Guangzhou China; ^3^ Department of Geriatrics National Key Clinical Specialty Guangzhou First People's Hospital South China University of Technology Guangzhou China; ^4^ Department of Respiratory and Critical Care Medicine Guangdong Provincial People's Hospital Guangdong Academy of Medical Sciences Guangzhou China; ^5^ Guangzhou National Laboratory Guangzhou China

**Keywords:** Bronchiectasis, COPD, COPD‐Bronchiectasis association, exacerbation, metabolome, microbiota

## Abstract

Bronchiectasis frequently co‐exists with chronic obstructive pulmonary disease (COPD‐bronchiectasis association [CBA]). We compared the microbiota and metabolome of bronchiectasis with (BO) and without airflow obstruction (BNO), COPD, and CBA. We determined how microbiota compositions correlated with clinical characteristics and exacerbations of CBA. We prospectively recruited outpatients with BNO (*n* = 104), BO (*n* = 51), COPD (*n* = 33), and CBA (*n* = 70). We sampled at stead‐state and exacerbation, and profiled sputum microbiota via 16S rRNA sequencing and metabolome via liquid chromatography/mass spectrometry. Sputum microbiota and metabolome profiles of CBA separated from COPD (*P* < 0.05) but not bronchiectasis, partly driven by Proteobacteria enrichment in CBA. An increasing microbial interaction but not microbiota compositions were identified at exacerbation. Pseudomonadaceae‐dominant CBA yielded lower Shannon–Wiener diversity index (*P* < 0.001), greater bronchiectasis severity (*P* < 0.05) and higher future exacerbation risk (HR 2.46, 95% CI: 1.34–4.52, *P* < 0.001) than other genera‐dominant CBA. We found a clear metabolite discrimination between CBA and COPD. Most of up‐regulated metabolites identified in CBA, were amino acids metabolites, which indicated that the accumulation of amino acids metabolites was related to the alteration of airway microbiota. To conclude, airway structural changes, but not airflow limitation, correlate more profoundly with microbiota and metabolome profiles (e.g. partly via Pseudomonadaceae‐amino acids metabolism links), shaping clinical outcomes of CBA.

## Graphical abstract

This study aimed to determine how microbiota compositions (via 16S rRNA sequencing) correlate with clinical outcome and metabolome (via liquid chromatography/mass spectrometry) of CBA. Sputum microbiome and metabolome profiles of CBA were well separated from COPD but not bronchiectasis, partly driven by the higher abundance of Proteobacteria in CBA. The Pseudomonadaceae‐dominant CBA yielded lower Shannon‐Wiener Diversity Index, greater bronchiectasis severity and higher risk of future exacerbations than other genera‐dominant CBA. The genera with differential abundance exhibited significant correlations with differential metabolites, indicating that microbiota and metabolome profiles (e.g. partly via Pseudomonadaceae‐amino acids metabolism links) may shape the clinical outcomes of CBA.

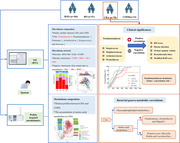

**Abbreviations**
BNO: bronchiectasis without airflow obstruction
BO: bronchiectasis with airflow obstruction
CBA: COPD‐bronchiectasis association
COPD: chronic obstructive pulmonary disease
FDR: false discovery rate
FEV_1_/FVC: ratio of forced expiratory volume in 1 s and forced vital capacity
HRCT: high‐resolution computed tomography
LDA: linear discriminant analysis
LEfSe: Linear discriminant analysis Effect Size analysis
OPLS‐DA: Orthogonal partial least‐squares discriminant analysis
PCoA: principal coordinate analysis
PERMANOVA: permutational multivariate analysis‐of‐variance
SWDI: Shannon‐Wiener Diversity Index

## 1 Introduction

Chronic obstructive pulmonary disease (COPD) and bronchiectasis are common chronic respiratory diseases associated with substantial healthcare burden worldwide [1, 2, 3]. The diagnosis of COPD has been based on incompletely reversible airflow obstruction associated with pollutant exposure [4], and bronchiectasis on pathological bronchial dilatation on chest radiography [5]. However, they have shared clinical manifestations and pathophysiology, including chronic airway inflammation, infection, and remodeling [6]. Clinically, they frequently co‐existed—COPD‐bronchiectasis association (CBA). The prevalence of CBA ranged from 4% to 75% among COPD patients and from 8.8% to 32% among bronchiectasis patients [7]. Compared with COPD or bronchiectasis, CBA yielded greater symptom burden, frequency and severity of exacerbations, and mortality risk [8, 9].

Dysbiosis is a hallmark of COPD and bronchiectasis. In bronchiectasis, decreased bacterial diversity correlated with poorer lung function, frequent severe exacerbations, and higher mortality [10]. Dysbiosis correlated with distinct clinical phenotypes and inflammatory endotypes in COPD [11, 12]. Both COPD and bronchiectasis are punctuated by recurrent exacerbations [13, 14]. The microbiota could shape the airway microenvironment via metabolites which are implicated in inflammatory signaling or immunomodulation [15, 16]. Profiling airway microbiota and metabolites would help appreciate the pathophysiology among CBA, COPD and bronchiectasis, and microbial interactions at exacerbations compared with steady‐state.

We sought to determine the microbiota compositions among bronchiectasis, CBA and COPD, and the association with the metabolome and clinical outcomes in CBA. Because the exposure to pollutants was mandatory to fulfill the definition of CBA [7], and because some patients had airflow limitation without pollutant exposure history (bronchiectasis with airflow obstruction, BO), we further compared microbiota and metabolome profiles between BO and CBA.

## 2 Methods

### 2.1 Study Population

In this prospective study, we recruited adults with clinically significant bronchiectasis and COPD [all underwent chest high‐resolution computed tomography (HRCT) and spirometry, when clinically stable, from outpatient clinics between March 2017 and December 2022. COPD denoted the post‐bronchodilator ratio of forced expiratory volume in 1 s and forced vital capacity (FEV_1_/FVC) <0.70, plus an exposure history to cigarette smoke (more than 10 pack‐years), biomass or heavy air pollution [17]. Bronchiectasis denoted the HRCT evidence of bronchiectasis (broncho‐arterial ratio >1, lack of tapering, and/or airway visible within 1 cm of pleura) compatible with respiratory symptoms [18]. CBA was diagnosed based on the radiology, airflow obstruction, symptom and exposure (ROSE) criteria [7].

Patients with bronchiectasis were stratified into BO (FEV_1_/FVC <0.70) and BNO (FEV_1_/FVC ≥0.70). At enrollment, patients remained stable and were free from upper respiratory infections or antibiotics therapy (apart from low‐dose macrolides) for >4 weeks. Exclusion criteria comprised active allergic bronchopulmonary aspergillosis or non‐tuberculous mycobacteria infection needing therapy, malignancy, pregnancy or lactation. The Ethics Committee of The First Affiliated Hospital of Guangzhou Medical University (Medical Ethics 2012 the 33th; Medical Ethics 2020, the 156th) gave protocol approval. All patients signed informed consent.

### 2.2 Study Design

Patients provided paired sputum and blood at enrollment. Clinical assessments included medical history inquiry, spirometry, quality‐of‐life (including COPD Assessment Test) [19] and radiologic assessment (e.g. modified Reiff score for HRCT [20]).

Patients were followed‐up at 3–6 months intervals and contacted investigators upon significant deterioration. Exacerbation visits were scheduled at onset of exacerbations, and sampled before antibiotic therapy where possible, or otherwise within 48 h after initiating antibiotic therapy. Sputum and blood were obtained at steady‐state and exacerbation visit. Bronchiectasis exacerbation denoted significant deterioration of three or more key symptoms persisting for >48 h, which required immediate changes in treatment [13]. For COPD and CBA, exacerbation denoted acute significant worsening resulting in immediate additional therapy [17].

### 2.3 Sputum Collection

After mouth rinsing, patients forcefully expectorated sputum into sterile containers. For patients who cannot spontaneously expectorate, we applied induction with 3% hypertonic saline. Following quality inspection, sputum was split for bacterial culture and stored in ‐80°C freezers. Supernatant was obtained after rinsing with phosphate buffer solution before metabolomic profiling (see Supporting information).

### 2.4 Sequencing Data Analytical Pipelines

The detailed procedure for DNA extraction, PCR amplification and sequencing analysis is provided in the Supporting information. DNA were extracted from sputum, followed by agarose gel electrophoresis and quality‐control with ND‐100 Nanodrop system. Quality‐filtered samples were subject to library construction. The V3‐V4 region of 16S ribosomal RNA was sequenced using Illumina Nova 6000 (Guangdong Magigene Biotechnology Co., Ltd., China). Sequences were deposited in GenBank (PRJNA1007275). All 16S rRNA gene data sets were performed using a standardized pipeline in QIIME 2.0 (Quantitative Insights Into Microbial Ecology 2.0) [21]. The demultiplexed sequencing reads were denoised to generate amplicon sequence variants (ASVs) using divisive amplicon denoising algorithm 2 (DADA2) with default parameters [22]. A custom Naive Bayes classifier was trained on Greengenes Database (Second Genome, Inc.) 13_8 99% operational taxonomic units to assign a taxonomy for the ASVs in the dataset. The samples were rarefied to 32,137 reads.

Alpha diversity was estimated by Shannon–Wiener diversity index (SWDI) and, and beta diversity by weighted Unifrac dissimilarity and visualized with principal coordinate analysis (PCoA). Permutational multivariate analysis‐of‐variance (PERMANOVA) was used to calculate the total distance between individuals within each group. Linear discriminant analysis Effect Size (LEfSe) was analyzed to assess the importance of taxon contributing to between‐group differences, with the significance threshold of LDA being set at >2.0. Significant inter‐genera Sparse Correlations for Compositional data (SparCC [23]) correlation coefficients >0.3 (false discovery rate [FDR]‐adjusted *P* < 0.05) were displayed with microbial co‐occurrence network graphics using the Gephi software.

The generated ASVs table was imported into the PICRUSt2 [24] and the Kyoto Encyclopedia of Genes, and the Genomes (KEGG) [25] database was used to predict the functional gene content of the various microbial communities represented in the Greengenes database for 16S rRNA gene sequences.

### 2.5 Widely Targeted Metabolomics

Sputum supernatant was employed for characterization of metabolomics with the widely‐targeted metabolomics (WT‐Met) approach, which is a large‐scale metabolite identification and accurate quantification system that combines the strengths of targeted and untargeted metabolomics technologies [26]. Briefly, high‐resolution mass spectrometry was adopted to conduct non‐targeted analysis (UPLC‐QTOF/MS analysis) using the mixed sample with data‐dependent acquisition mode, followed by comparison with both the public database (including METLIN, HMDB, KEGG database) and self‐built database (MWDB, Wuhan MetWare Biotechnology Co., Ltd.) to build a new project‐specific database. Next, the multiple reaction monitoring mode of triple quadrupole mass spectrometry was performed for precise quantification of compounds within the samples. See Supporting information for the detailed experimental procedure.

### 2.6 Statistical Analysis

Data were processed with R version 4.2.2, python 3.10 and GraphPad Prism (version 8.0.1 GraphPad Inc., San Diego, USA). Categorical data were analyzed by determining the frequency and percentage, whereas continuous data using mean ± standard deviation or median and interquartile range (IQR). Associations between microbial variables and clinical parameters were assessed using Spearman's correlations, and between‐group differences using analysis‐of‐variance, Kruskal‐Wallis test, or Wilcoxon matched‐pair signed‐rank test. Multiple comparisons were made by applying Bonferroni's corrections for homoscedastic and Tamhane corrections for heteroscedastic data. Microbial characterization was determined by LEfSe with Wilcoxon rank‐sum test and linear discriminant analysis (LDA)‐score (log_10_) >2 (permutation‐based FDR‐*P* <0.05). For significant between‐group differences in microbial/metabolomic profiles in PCA, we applied Orthogonal partial least‐squares discriminant analysis (OPLS‐DA) to highlight differentially expressed metabolites. We conducted redundancy analysis, which determined the interrelationship between multivariable datasets and explanatory variables, to analyze the impact of key clinical parameters on microbiota compositions. We evaluated the correlations between the genera and clinical parameters and metabolites by Spearman's correlation (FDR‐*P* <0.05). LEfSe analysis, PCoA and taxa‐metabolites correlation analysis was performed using the OmicStudio tools [27] at https://www.omicstudio.cn/. Detailed analysis procedures were provided in the Online Supplement or deposited in GitHub (https://github.com/Dr‐Hezf/CBA‐microbiota.git).

## 3 Results

### 3.1 Patient Characteristics and Sample Quality

**FIGURE 1:**
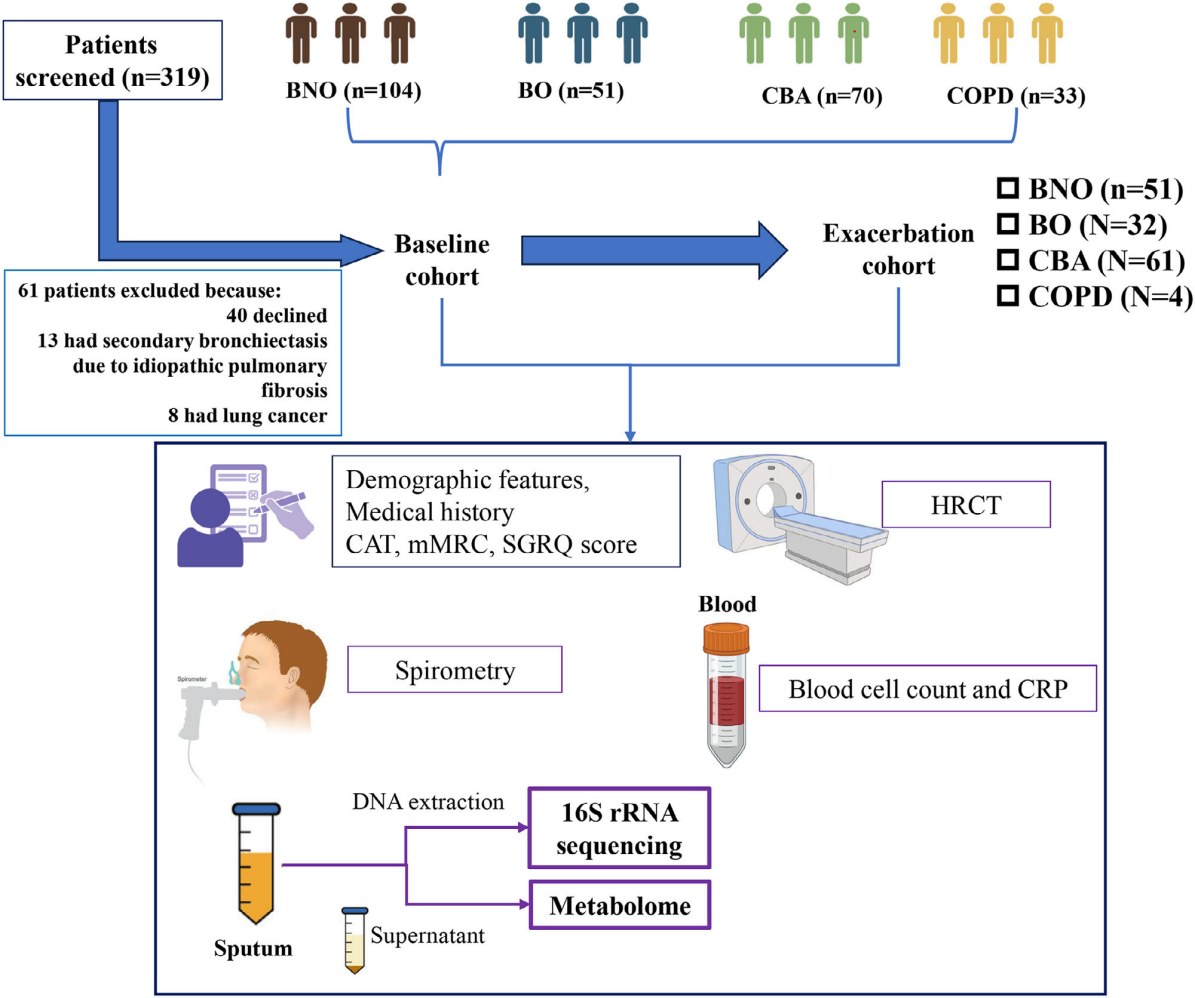
A schematic illustration of the overall study design and patients recruited.

Of 319 patients screened, 258 were recruited and followed‐up for >6 months. 104 had BNO, 51 had BO, 70 had CBA, and 33 had COPD. All patients provided sputum at initial stable visits (baseline cohort, Figure 1), of whom 19 underwent sputum induction (3 BNO, 1 BO, 3 CBA, and 12 COPD) because of the failure to produce spontaneous sputum. 395 steady‐state and 148 exacerbations visits yielded sufficient sputum for sequencing, with a median (IQR) of 1 (1–2) steady‐state and 1 (1–2) exacerbation samples per patient.

Patients with COPD were mostly males and older, and mostly had smoking exposure. The proportion of patients receiving inhaled corticosteroids and inhaled bronchodilator increased from BNO, BO, CBA to COPD. Patients with CBA had higher sputum volume, greater lung function impairment (except for FEV_1_). These characteristics, however, did not differ between CBA and BO (Table 1).

**TABLE 1:** Demographic and clinical characteristics of study participants.

Parameters	BNO (*N* = 104)	BO (*N* = 51)	CBA (*N* = 70)	COPD (*N* = 33)	*p* value^$^ (CBA vs BNO)	*p* value^$^ (CBA vs BO)	*p* value^$^ (CBA vs COPD)
**Age (year)**	49 ± 13	46 ± 14	54 ± 12	65 ± 8	0.04	<0.001	<0.001
**BMI (kg/m^2^)**	19.9 ± 4.9	20.3 ± 3.1	21.4 ± 3.2	22.4 ± 3.0	0.013	ns	ns
**Gender (% female)**	71 (68.3%)	32 (62.7%)	31(44.3%)	0 (0%)	<0.05	ns	<0.05
**Smoking status** ^†^					<0.05	<0.05	<0.05
Never‐smokers (No., %)	94 (90.4%)	48 (94.1%)	45 (64.3%)	0			
Ex‐ or current‐smokers (No., %)	10 (9.6%)	0	25 (35.7%)	33 (100%)			
**Exposure of biomass fuel** ^††^	68 (65.4%)	0	55 (78.6%)	12 (36.4%)	<0.05	<0.05	<0.05
**Disease duration (year)** ^‡^	17 ± 10	22 ± 12	19 ± 13	12 ± 8	ns	ns	ns
**24‐h sputum volume (ml)**	25 (10, 50)	30 (20, 50)	30 (20, 50)	5 (5, 10)	ns	ns	<0.001
**FEV_1_ (L)**	1.91 ± 0.70	1.41 ± 0.53	1.32 ± 0.56	1.33 ± 0.62	<0.001	ns	ns
**FEV_1_% predicted**	73.78 ± 16.68	49.86 ± 16.48	48.12 ± 16.96	47.13 ± 19.30	<0.001	ns	ns
**FVC(L)**	2.59 ± 0.75	2.46 ± 0.76	2.35 ± 0.77	2.80 ± 0.77	ns	ns	0.024
**FVC% predicted**	80.00 ± 18.49	71.99 ± 19.97	70.47 ± 16.97	81.20 ± 18.89	0.006	ns	0.034
**FEV_1_/FVC (%)**	77.43 ± 8.78	58.46 ± 8.11	55.32 ± 10.67	44.95 ± 12.18	<0.001	ns	<0.001
***PA* colorization, n (%)** ^‡‡^	28 (26.9%)	27 (52.9%)	30 (42.8%)	—	—	—	—
**Exacerbation frequency in the past year**	1 (1,2)	1 (1, 2)	1 (1,3)	1 (0,1)	ns	ns	<0.001
**CAT score**	13.3 ± 7.0	14.4 ± 7.6	13.3 ± 7.5	10.7 ± 5.9	ns	ns	ns
**SGRQ score**	27.2 ± 16.9	34.1 ± 15.5	33.5 ± 18.6	33.3 ± 16.1	ns	ns	ns
**Bronchiectasis severity index**	4.6 ± 3.2	7.9 ± 4.5	8.1 ± 3.8	—	<0.001	ns	—
**HRCT Reiff score**	7.6 ± 4.5	8.5 ± 3.9	9.0 ± 4.7	—	ns	ns	—
**Medication at enrolment**							
Inhaled corticosteroids (No., %)	8 (7.7%)	6 (11.8%)	20 (28.6%)	21 (63.6%)	<0.001	0.045	0.001
Low‐dose macrolides (No., %)	9 (8.7%)	6 (11.7%)	8 (11.4%))	0	ns	ns	ns
Inhaled bronchodilator (No., %)	37 (35.6%)	29 (58.0%)	62 (88.6%)	33 (100%)	<0.001	<0.001	ns

Abbreviations: BNO = Bronchiectasis without airflow obstruction, BO = Bronchiectasis with airflow obstruction, CBA = COPD‐Bronchiectasis association, COPD = Chronic obstructive pulmonary disease, BMI = body mass index, FEV_1_ = The forced expiratory volume in a 1‐s, FVC = forced vital capacity, PA = *Pseudomonas aeruginosa*, CAT = COPD assessment test, SGRQ = St George's Respiratory Questionnaire, HRCT = high resolution computerized tomography.

^†^ Patient demographic data at baseline, ex‐ or current‐smoker had at least 10 pack‐years.

^††^ Patient demographic data at baseline, defined as with biomass exposure for at least 10 years.

^‡^ Patient demographic data at baseline, defined as the time from the first respiratory symptom onset.

^‡‡^ Defined as the appearance of positive culture of PA at least 2 occasions at least 3 months apart within 1 year.

^$^ Multiple comparisons were performed with the Bonferroni correction for homoscedastic data and Tamhane correction for heteroscedastic data.

We obtained 81,769,907 high‐quality 16S rRNA reads [median: 113,118 (range: 32,137–252,716) per sample], with 305 ASVs identified at genus. Rarefaction curve indicated sufficient quality for analyses (Figure S1, Supporting information).

### 3.2 Differential Microbiota Compositions at Steady‐State across all Groups

**FIGURE 2:**
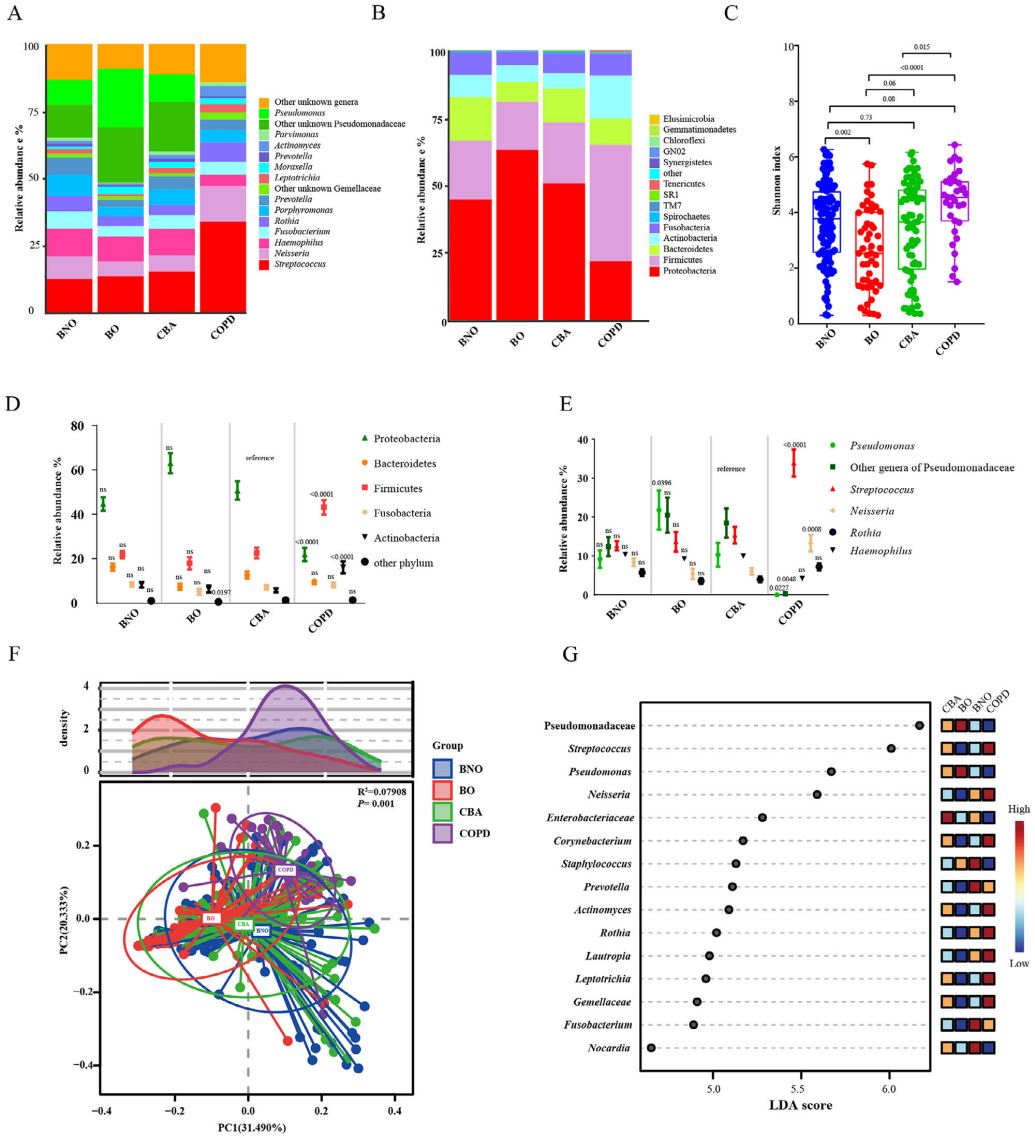
Baseline sputum microbiota profiles across the four groups. Composition of the major phyla (A) and genera (B), and alpha diversity (ShannonWiener diversity index) at baseline. The relative abundance of microbiota is shown at phylum (D) and genera (E) levels at baseline. (F) Principal coordinate analysis based on the weighted Unifrac dissimilarity for four groups. (G) LEfSe showing the genera enriched in each group (LDA >2.0, FDR‐P <0.05). *P < 0.05, **P < 0.01, ***P < 0.001, ns: no significant difference. BNO: bronchiectasis without airflow obstruction; BO: bronchiectasis with airflow obstruction; CBA: COPD‐bronchiectasis association; LDA: Linear discriminant analysis; LEfSe: Linear discriminant analysis effect size; FDR: false‐discovery rate. Note: Unknown *Pseudomonaceae* (other genera in *Pseudomonaceae*) and *Pseudomonas* were displayed separately herein but subsequently combined in further analysis.

At phyla level, Proteobacteria, Firmicutes, and Bacteroidetes dominated in BNO, BO and CBA, while Firmicutes, Proteobacteria and Actinobacteria dominated in COPD at steady‐state. The genera *Pseudomonadaceae, Streptococcus*, and *Haemophilus* dominated in CBA, BO and BNO, while *Streptococcus, Neisseria* and *Rothia* dominated in COPD (Figure 2A,B). COPD yielded the highest SWDI, followed by BNO, CBA and BO (mean: 4.34 vs 3.62 vs 3.38 vs 2.69, *P* < 0.001) (Figure 2C). CBA exhibited a markedly higher relative abundance of Proteobacteria and *Pseudomonadaceae*, and significantly lower relative abundance of Firmicutes, *Actinobacteria*, *Streptococcus* and *Neisseria* compared with COPD (both *P* < 0.05). Furthermore, CBA yielded markedly lower SWDI compared with COPD (*P* < 0.05, Figure 2D,E). PCoA plots based on weighted Unifrac distance revealed significant inter‐group separation (PERMANOVA *R* ^2^ = 0.079, *p* = 0.001), particularly for COPD vs. other groups (Figure 2F). LEfSe analysis showed that *Streptococcus, Neisseria*, *Corynebacterium*, *Actinomyces*, and *Rothia* were enriched in COPD while Enterobacteriaceae in CBA (Figure 2G). To identify confounding factors of microbiota compositions, Figure S2, Supporting information displays the subgroup comparisons based on sputum sampling method (induced vs spontaneous sputum), sex, medication exposure and smoking status.

### 3.3 Clinical Features Correlates of Sputum Microbiota at Steady‐State of CBA

**FIGURE 3:**
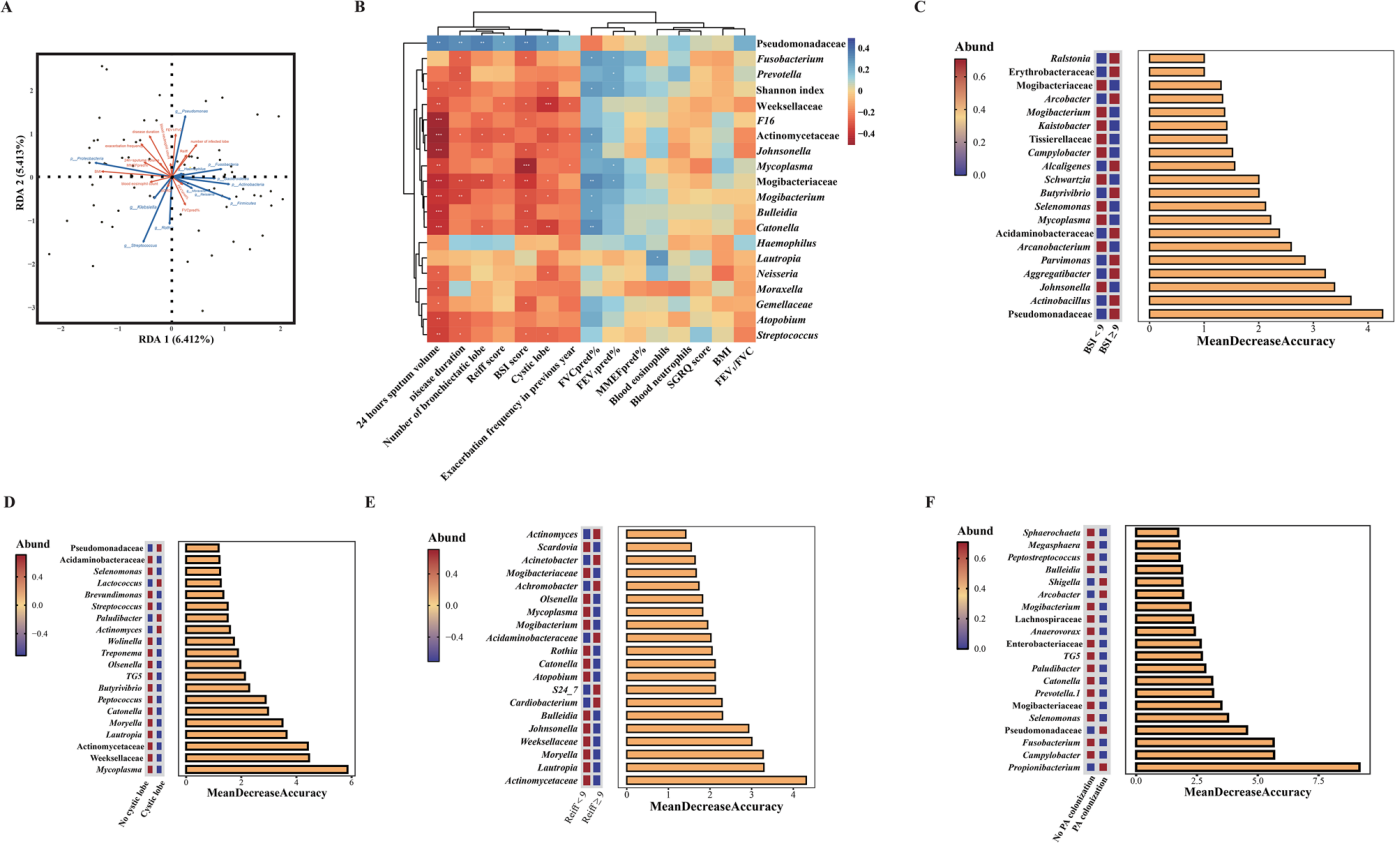
The association of sputum microbiota and the clinical characteristics in patients with CBA. (A) Redundancy analyses of the microbiota compositions in CBA. Each node represents an individual sputum sample. Blue labels and arrows indicate microorganisms, red labels and arrows indicated clinical factors. The length of the arrow indicates the intensity of impact of clinical factors on microbial compositional changes. Originating from the central point, the clinical factors correlate positively with the microorganisms if their arrows point to the same direction, and the vice versa. The impact of each clinical factor was tested by envfit analysis and the overall impact tested by permutation test. Further details are displayed in Table S1, Supporting information. (B) Heatmap showing Spearman's correlations between the genus and clinical parameters in CBA. *P < 0.05, **P < 0.01, ***P < 0.001. Random forest plot was conducted to identified key taxa between different clinical feature (C) for BSI score, (D) for the presence of cystic lobe, (E) for Reiff score and (F) for the presence of Pseudomonas aeruginosa colonization. V1: day 1 (baseline steady‐state); A1: day 1 upon exacerbation; PCoA: Principal coordinate analysis; CBA: COPD‐bronchiectasis association; LDA: Linear discriminant analysis; LEfSe: Linear discriminant analysis effect size; FDR: false‐discovery rate; PC1: the first principal component; ASV: generate amplicon sequence variant.

Redundancy analysis revealed any particular genera may correlate with the core clinical characteristics at steady‐state of CBA (Figure 3A, Table S1, Supporting information). The Shannon index exhibited a modestly positive association with FEV_1_% predicted and FVC% predicted, and a negative association with the disease duration and 24‐h sputum volume. The relative abundance of Pseudomonadaceae positively correlated with the BSI score, disease duration, 24‐h sputum volume, the number of bronchiectatic lobes and modified Reiff score; whereas the relative abundance of *Streptococcus*, Mogibacteriaceae, Actinomycetaceae, and Weeksellaceae correlated negatively with these clinical parameters (Figure 3B, correlation coefficient and *p* values were deposited in GitHub dataset). Random Forest plot identified Pseudomonadaceae, *Actinobacillus*, *Mycoplasma*, and Actinomycetaceae as the key taxa in differentiating patients with CBA into different strata—disease severity, BSI score, cystic bronchiectasis and modified Reiff score. Moreover, *Propionibacterium*, *Campylobacter*, and *Fusobacterium* were influenced by *Pseudomonas aeruginosa* colonization. (Figure 3C–F)

### 3.4 Pseudomonadaceae Dominance and Clinical Outcomes of CBA

**FIGURE 4:**
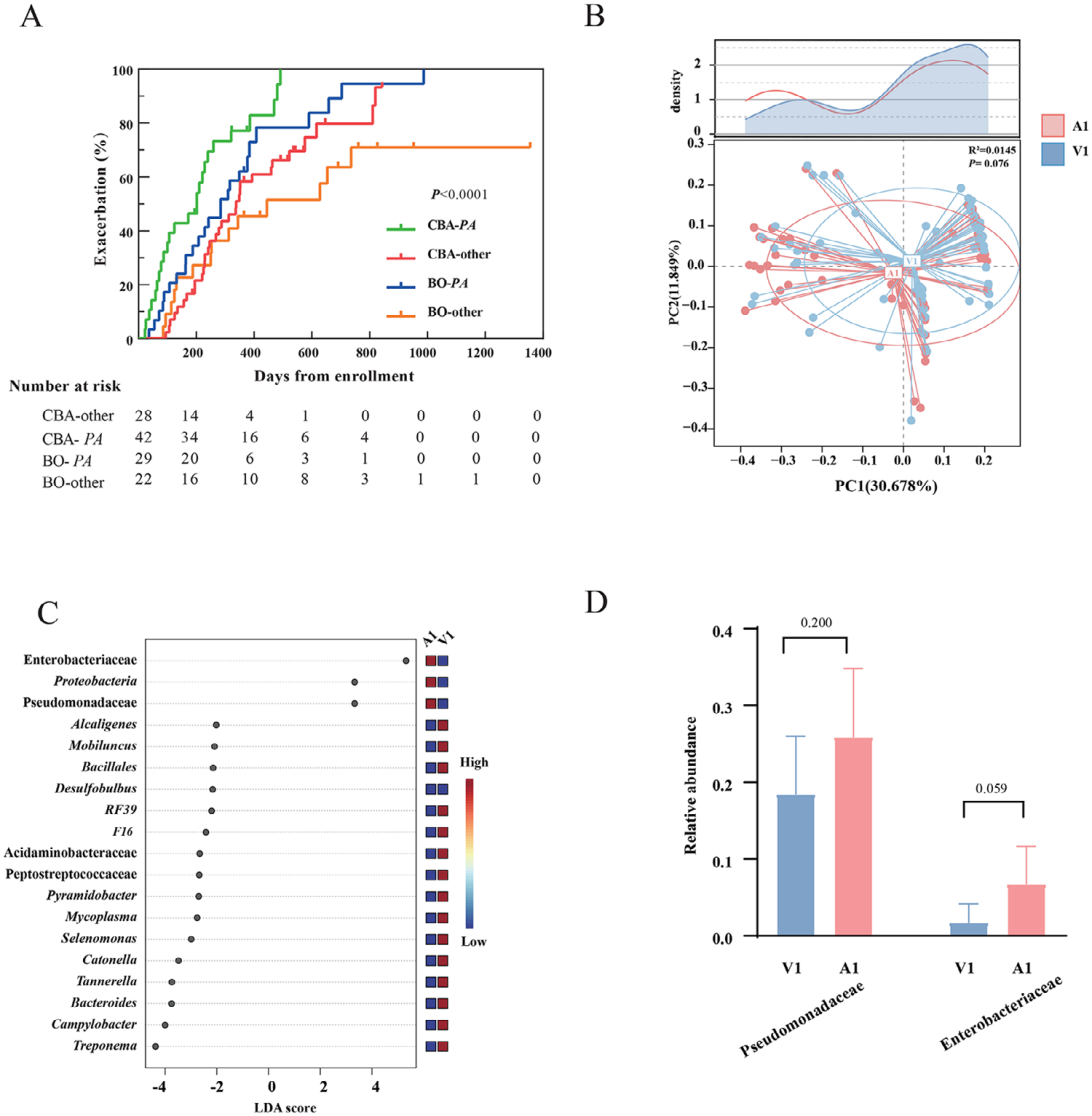
The association of sputum microbiota and future exacerbation risk in patients with CBA, and the comparison of microbial profiles between steady‐state and exacerbation. (A) Kaplan–Meier curves for the exacerbation based on the dominant organism (the highest ASV%) at baseline. (B) PCoA based on the weighted Unifrac value for sputum from steady‐state and exacerbation of CBA, with the density plot showing the distribution of PC1. (C) LEfSe analysis showing the enriched microbial genera (LDA >2.0, FDR‐P<0.05). (D) The average relative abundance is ranked in descending order (shown for each genus). The relative abundance of two key genera associated with exacerbation onset of CBA. BNO: bronchiectasis without airflow obstruction. BO: bronchiectasis with airflow obstruction. COPD: chronic obstructive pulmonary disease; CBA: COPD‐bronchiectasis association.

As mentioned above, Pseudomonadaceae correlated with the disease severity of CBA, and reportedly predisposed to clinical progression of bronchiectasis in COPD and airflow obstruction in bronchiectasis [8, 28]. We therefore next stratified, among CBA, steady‐state samples into Pseudomonadaceae‐dominant (Pseudomonadaceae being the highest ASV%, n = 28) and other genera‐dominant subgroups (other genera being the highest ASV%, *n* = 42). Pseudomonadaceae‐dominant subgroup yielded significantly lower SWDI and higher BSI (Figure S3 and Table S2, Supporting information). At follow‐up, the median duration of follow‐up was 187 (IQR 72–293) days for Pseudomonadaceae‐dominant subgroup and 234 (IQR 101–321) days for other genera‐dominant subgroup. Among patients who reported exacerbations during follow‐up (85.7% in Pseudomonadaceae‐dominant subgroup and 69.0% in other genera‐dominant subgroup), there was no significant difference in the median time to the first exacerbation in Pseudomonadaceae‐dominant subgroup (144 [IQR 62‐246] days vs. 195 [40‐318] days, *p* = 0.688). Pseudomonadaceae‐dominant subgroup had a markedly higher risk of experiencing exacerbations at follow‐up (HR 2.461, 95%CI: 1.341‐4.516, *P*<0.0001). Similar findings applied in patients with BO (other genera‐dominant vs Pseudomonadaceae‐dominant, HR:2.005, 95%CI:1.054‐3.816, *p* = 0.034). Notably, the Pseudomonadaceae‐dominant subgroup with CBA exhibited significantly higher exacerbation risk than those with BO (HR:1.818, 95%CI: 1.002‐3.298, *p* = 0.049) (Figure 4A, Table S3, Supporting information). The Cox regression analysis was performed to adjust for potential confounders such as BSI, CAT score, age, lung function, and the HRCT modified Reiff score (Table S4, Supporting information).

### 3.5 Differential Microbiota Compositions at Exacerbation Across All Groups

Among the exacerbation visits, 148 patients (51 BNO, 32 BO, 61 CBA and 4 COPD) provided sputum for 16S rRNA sequencing. Similar with steady‐state, BNO, BO and CBA yielded greater similarity in microbiota compositions, which diverged with those of COPD at exacerbation. LEfSe analysis indicated enrichment of *Streptococcus, Neisseria*, and *Rothia* in COPD, *Enterobacteriaceae* in CBA, *Pseudomonas* and *Staphylococcus* in BO, and *Prevotella* and *Bacteroides* in BNO. (Figure S4, Supporting information)

Microbiota compositions were similar between steady‐state and exacerbation in CBA (*p* = 0.076) (Figure 4B). Both *Pseudomonas* and *Enterobacteriaceae* tended to be associated with exacerbation onset (LDA score >4), with only numerical differences in the relative abundance between steady‐state and exacerbation (Figure 4C,D).

### 3.6 Microbial Interactions at Steady‐State and Exacerbations Across All Groups

**FIGURE 5:**
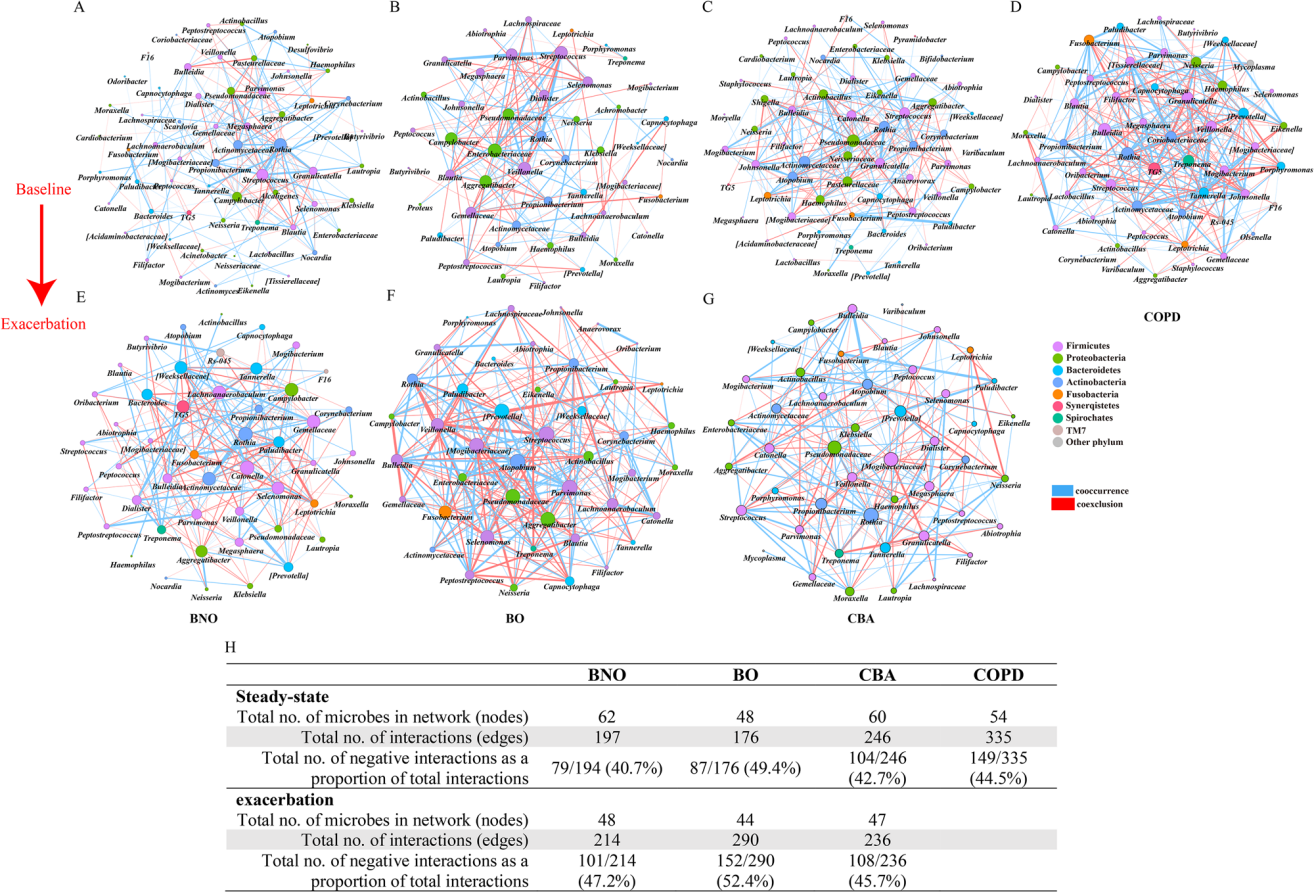
Co‐occurrence analysis of the microbiota across the four groups. Microbial co‐occurrence network of BNO (A, B), BO (C, D), CBA (E, F) and COPD (G) from baseline to exacerbation onset, and a summary table listing the network characteristics (H). Each node represents a genus. Nodes were colored based on the phylum classification. The size of the node is proportional to the number of interactions. Each edge represents a significant SPARCC correlation between the pairs of nodes (FDR‐P <0.05). The width of the edge is proportional to the absolute correlation coefficient. Edges are colored based on co‐exclusion (red) or co‐occurrence (blue) relationship. The significant inter‐genera correlation coefficients >0.3 (false discovery rate [FDR]‐adjusted *P* < 0.05) are displayed. BNO: bronchiectasis without airflow obstruction; BO: bronchiectasis with airflow obstruction; CBA: COPD‐bronchiectasis association; COPD: chronic obstructive pulmonary disease; FDR: false‐discovery rate.

Because no significant difference was observed between the stable‐state and exacerbation in terms of microbiota composition and the relative abundance, we further explored the alteration of the interaction among different microbes. In the microbiota co‐occurrence network (Figure 5), the number of correlation pairs during exacerbations were similar with that at the stable stages, across BNO, BO, and CBA. In terms of the number of microbes, the three groups exhibited varying degrees of decline, with BNO showing the most significant reduction from stable‐state to exacerbation onset. All three groups—BNO, BO, and CBA—showed a higher proportion of co‐exclusion (negative interaction) relationships at exacerbation onset, with BNO showing the most pronounced changes.

The Proteobacteria (particularly Pseudomonadaceae) was the key taxa in the network in CBA and BO, but not in BNO and COPD. In CBA, Pseudomonadaceae, *Streptococcus* and *Rothia* were the key genera exhibiting significant interactions with other microbes at stead‐state; however, at exacerbation onset Pseudomonadaceae, *Moraxella*, and *Klebsiella* exhibited negative correlations with other microbes.

### 3.7 Prediction of Microbial Community Function

**FIGURE 6:**
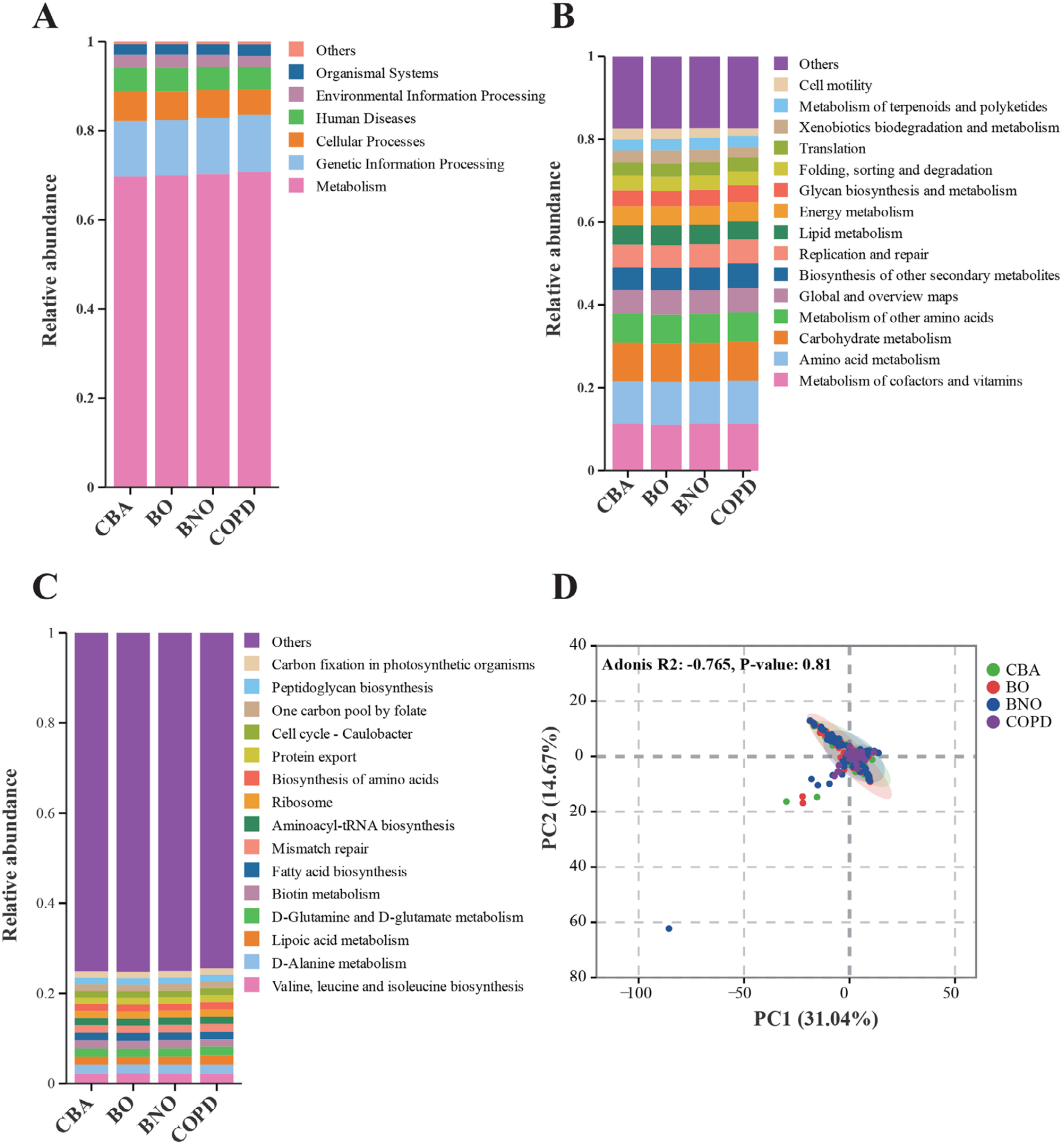
Prediction of microbial community function. The relative abundances of enriched (A) KEGG Pathways Level 1. (B) KEGG Pathway Level 2 and (C) KEGG Pathway Level 3 in different BNO, BO, CBA, and COPD. (D) Principal component analysis for microbial community function across four groups.

Both the PICRUSt2 and the KEGG database were utilized to explore the microbial community function. Metabolism (∼70%) was the dominant function in KEGG pathway level 1, followed by genetic information processing (∼12%), cellular processes (∼6%), human diseases (∼5%), environmental information processing (∼3%%) and organismal systems (∼2.5%) (Figure 6A). Metabolism of cofactors and vitamins, Amino acid metabolism, and Carbohydrate metabolism were the top enriched functions, accounting for ∼30% across the four groups (Figure 6B). Additionally, “valine, leucine, and isoleucine biosynthesis”, “d‐alanine metabolism”, and “d‐glutamine and d‐glutamate metabolism” presented the top enriched function at KEGG level 3 (Figure 6C). No significant separation was found across BNO, BO, CBA, and COPD in the PCA analysis (Figure 6D).

### 3.8 Bacterial Genera‐Metabolite Interactions in CBA

Microbial metabolites reportedly conferred immunomodulatory effects on chronic respiratory diseases [29]. To ascertain the microbiota‐metabolite associations in CBA, we performed metabolomic analysis based on the paired available sputum from 241 patients (96 BNO, 48 BO, 66 CBA and 31 COPD, with one pair of samples per patient). We identified 1799 metabolites (Figure S5A, Supporting information). Consistent with microbial profiles, metabolomic composition in COPD clearly separated from other groups (BNO, BO and CBA almost overlapped) based on the first (explaining 29.5% of variance) and second principal component (explaining 4.5% of variance) (Figure S5B, Supporting information).

**FIGURE 7:**
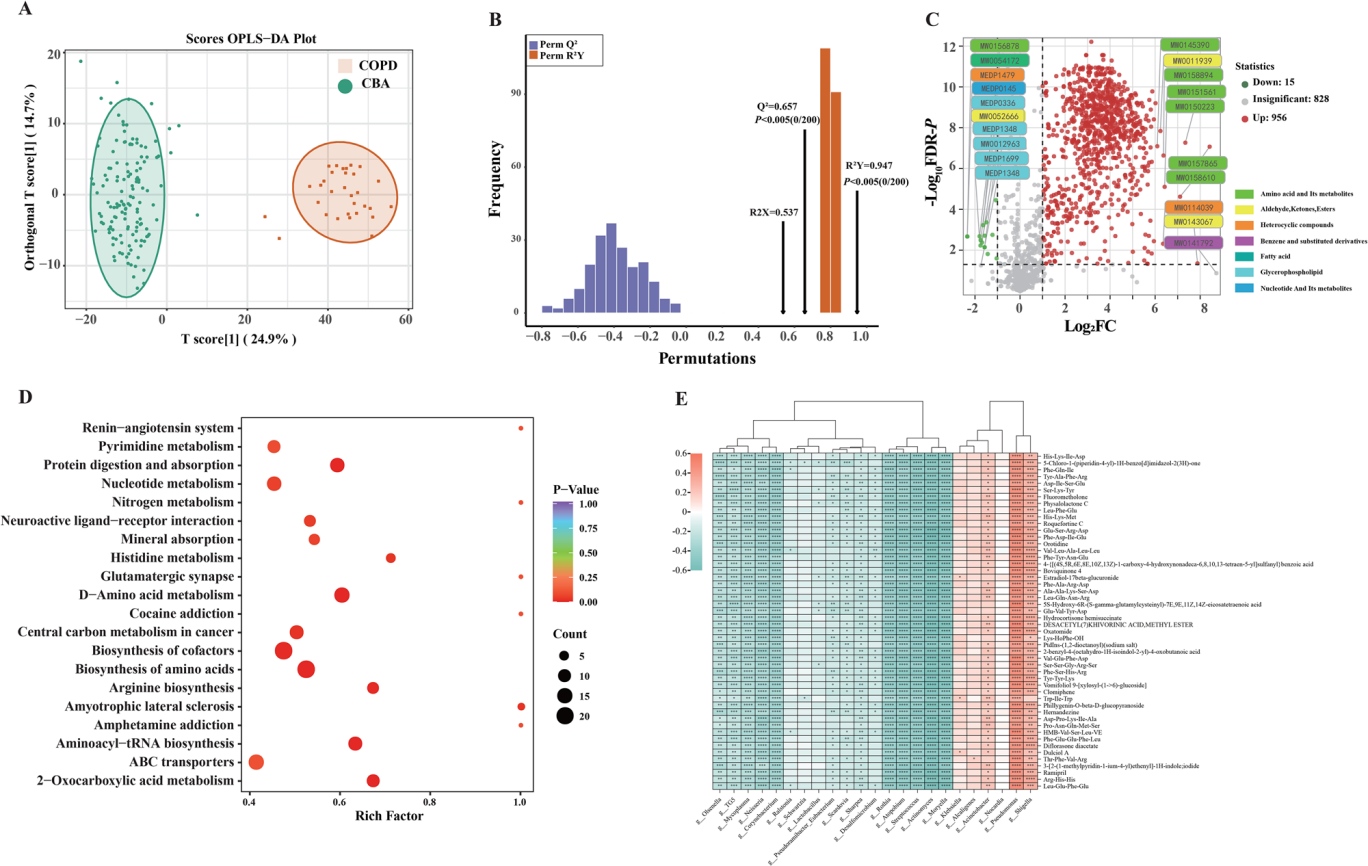
Differential sputum metabolomic features in CBA and COPD. (A) OPLS‐DA score plot. (B) OPLS‐DA validation plot (intercept: Q^2^ = 0.657, *P* < 0.005); (C) Volcano plot showing the differentially enriched [log2 (fold‐change) on *X*‐axis] and differentially expressed [−log10 (FDR‐P) on *Y*‐axis] metabolites in COPD and CBA. The green dots represent down‐regulated metabolites, while red ones represent up‐regulated metabolites. (D) Bubble plot of the top 20 enriched metabolic pathways. (E) Spearman's correlation analysis between the top differential taxa and metabolites. OPLS‐DA: orthogonal partial least squares‐discriminant analysis; FDR: false‐discovery rate; COPD: chronic obstructive pulmonary disease; CBA: COPD‐bronchiectasis association.

The PCoA and OPLS‐DA plots showed a clear inter‐group discrimination between CBA and COPD at steady‐state (Figure 7A,B, Figure S5C, Supporting information). The volcano diagram was used to screen out 971 differential metabolites (fold change >2 or <0.5, *P* < 0.05) identified in CBA (956 up‐regulated and 15 down‐regulated metabolites), 329 of which were mapped into 134 KEGG pathways. This included biosynthesis of cofactors (22) and amino acids (20) and d‐Amino acid metabolism (14) (Figure 7C, Figure S6 and S7, Supporting information). Almost half of the down‐regulated metabolites in CBA were classified into glycerophospholipid metabolism, which was reportedly linked to eosinophilic inflammation in COPD [30]. Among all the enriched pathways, amino acid metabolism‐related pathways were the most abundant. Tryptophan metabolism, reportedly conveying protective effect in lung function impairment [30], was heightened in CBA. The top 20 significantly enriched pathways are displayed in Figure 7D.

LEfSe analysis identified the taxa with the greatest differences in relative abundance between CBA and COPD. Twenty‐three genera with log_10_LDA scores greater than 2.0 were selected for correlation analysis with the top 50 differential metabolites (|r| > 0.4 and *P* < 0.05). Some amino acid metabolism such as leucine, tyrosine, lysine, increased significantly and correlatedly positively with *Pseudomonas, Acinetobacter*, and *Shigella*, but negatively with *Streptococcus*, *Moryella*, *Rothia* and *Lactobacillus*. (Figure 7E, Figure S6–S8, Tables S5 and S6, Supporting information).

## 4 Discussion

Despite similar microbiota and metabolome of CBA compared with BNO and BO, they separated from that of COPD. This was partly explained by the greater abundance of Proteobacteria in CBA. A change of microbial interactions but not microbiota compositions were identified at exacerbation compared with steady‐state across all groups. However, Pseudomonadaceae‐dominant CBA yielded lower SWDI, greater disease severity and higher risk of future exacerbations. These might be modulated by some differentially expressed metabolites, particularly the accumulation of amino acid metabolism.

Patients with CBA have been frequently excluded from conventional clinical studies; therefore, little is known regarding their pathophysiology compared with COPD and bronchiectasis. Previous studies have demonstrated airway dysbiosis in COPD and bronchiectasis [31]. There was a high relative abundance of Proteobacteria (particularly *Pseudomonas*) in bronchiectasis [10, 32] and *Haemophilus* and *Moraxella* in COPD [33]. Here, we focused on CBA which had the shared characteristics of COPD and bronchiectasis. Consistent with previous findings that *Pseudomonas* predicted poorer outcomes in bronchiectasis and COPD, we have unveiled the association of Pseudomonadaceae‐dominant microbial profiles and future exacerbation risks. The decreased microbial diversity arising from pulmonary structural damages might have driven the heightened chronic neutrophilic inflammation which could confer greater exacerbation risks [34, 35]. Because previous exacerbation was the strongest predictor of future exacerbation risks, the decreased microbial diversity could be the consequence of repeated antibiotics exposure due to frequent exacerbation. We should, however, remain cautious regarding the directionality of causative effects.

We have identified substantial similarity in microbial composition between BO and CBA. Considering the comparable airflow limitation between BO and CBA (which merely differed in pollutant exposures), airway destruction but not airflow limitation might have contributed more profoundly to dysbiosis in bronchiectasis. Both air pollutants and pathogens might have been implicated in airflow limitation and structural changes. However, the SWDI was lower and *Pseudomonas* was enriched in BO. This was not surprising, because chronic infection with *Pseudomonas aeruginosa* correlated with greater lung function impairment [36].

Consistent with the findings by Huang et al. [37], we showed a considerable overlap in the microbial composition among CBA, BNO and BO, and a clear separation from COPD. This reaffirmed that airway destruction elicited more profound impacts than airflow limitation on both the microbial composition (including their interactions) and metabolome. Congruent with this, decreased microbial diversity was reportedly associated with structural damage, but not FEV_1_%, in bronchiectasis [38]. In CBA, structural damage may correlate with FEV_1_% and FVC% predicted, but not FEV_1_/FVC.

Airway dysbiosis is closely associated with exacerbations. However, neither microbial composition nor diversity differed at exacerbation in CBA, which mirrored other reports of bronchiectasis [32, 39]. Both *Enterobacteriaceae* and *Pseudomonas* were associated with exacerbation (LDA >2), despite the nominal difference in their relative abundance. This highlighted the significance of detecting *Enterobacteriaceae* and *Pseudomonas* at exacerbation—the key predictors of hospitalization and mortality [40]. Furthermore, microbial diversity alone was insufficient to explain for exacerbation onset [32, 39]. Therefore, microbial interaction analysis was performed, revealing a higher proportion of negative interaction at exacerbation than steady‐state. This might help interpret the onset of exacerbation, as reported in the CAMER study [39]. Interestingly, in the CAMER cohort, patients had better lung function (FEV_1_%pred: 74 [54–87]%) but more severe disease (BSI: 9 [6–13]) [41]. This led us to further consider whether the changes in microbial relationships played a greater role in eliciting acute exacerbations in BNO prior to the development of airflow obstruction (i.e. before progression to BO or CBA). When airflow limitation developed, dysbiosis might play a less prominent role in triggering exacerbation as compared with immune dysregulation and aggravated inflammation. This aligned with the previous studies showing that macrolides reduced the risk of acute exacerbations in bronchiectasis patients through immune modulation rather than direct antimicrobial effects [42]. Whether different subtypes of bronchiectasis were triggered by distinct factors may warrant further research with larger sample sizes.

The greater similarity in microbial composition and metabolites between bronchiectasis and CBA (separated from COPD) might justify a bronchiectasis‐centered therapeutic strategy towards CBA. Similar with bronchiectasis, clinicians should be vigilant to *Pseudomonas* detection in CBA. Our study highlighted the complexity of microbe‐metabolite association. Indole‐3‐acetamide secretion was driven by *Lactobacillus*, *Streptococcus Neisseria* (lower abundance in CBA) and *Pseudomonas aeruginosa* (higher abundance in CBA) [30]. The increased indole‐3‐acetamide levels in CBA might be associated with greater lung function impairment (FEV_1_/FVC), or enrichment of *Pseudomonas* as compared with COPD.

Some limitations should be acknowledged. Because our cohort initially focused on bronchiectasis, we recruited COPD patients as disease controls since 2021 to investigate bronchiectasis overlap syndromes. We did not include healthy controls and never‐smoker COPD patients with biomass exposure as a comparator group. Because no prior evidence pertaining to the differences in microbial/metabolite compositions was available, we did not estimate sample sizes. Oral contamination cannot be precluded; however, efforts have been made to secure sputum quality. Sputum collected at COPD exacerbation was limited, constraining our interpretation regarding microbial interactions. Less than 10% of sputum was derived from induction; however, previous studies and our findings suggested non‐significant impact on microbial compositions [12]. We did not detect bacterial load which would predict therapeutic responses [43]. Furthermore, there was no sampling of environmental control or secretions from upper airways.

In this prospective cohort study, CBA exhibits a greater similarity in microbiota and metabolomic compositions with bronchiectasis but not COPD. The change of microbe's interaction may contribute to exacerbations in CBA. The association between microbiota and clinical outcomes of CBA might be partly explained by the microbiota‐metabolite associations.

## Author Contributions

Zhen‐feng He and Wei‐jie Guan drafted the manuscript; Zhenfeng He, Wei‐jie Guan and Nan‐shan Zhong contributed to conception and design; Zhen‐feng He, Xiao‐xian Zhang, Cui‐xia Pan, Yan Huang, Chun‐lan Chen, Shan‐shan Zha, Lai‐jian Cen, Hui‐min Li, Zhen‐hong Lin and Sheng‐zhu Lin were responsible for patient recruitment and data collection; Zhen‐feng He, Xin‐zhu Yi, Han‐qin Cai, Lei Yang, Jia‐qi Gao, and Zhang Wang performed data analysis; Wei‐jie Guan, Zhang Wang and Nan‐shan Zhong critically revised the manuscript. All authors have approved the final submission.

## Conflicts of Interest

The authors declared no conflicts of interest.

## Supporting information



Supporting Information
