# Supplementary material for: Sputum Microbiota Compositions Correlate With Metabolome and Clinical Outcomes of COPD‐Bronchiectasis Association: A Prospective Cohort Study
Source: Exploration (Beijing). 2025 May 4;5(4):e20240149. doi: 10.1002/EXP.20240149 (PMC12380057; doi:10.1002/EXP.20240149)
Supplement: Supplementary file 1 — Supporting Information [file EXP2-5-e20240149-s001.docx]

**Supplementary materials**

**Sputum microbiota compositions correlate with metabolome and clinical outcomes of COPD-bronchiectasis association: A prospective cohort study**

**Methods**

***Sputum collection***

Spontaneous or induced sputum was obtained from all recruited subjects and quality controlled upon collection. Briefly, sputum plugs that contained the most viscous material were placed in a Petri dish and isolated from saliva. Sputum specimens with a squamous epithelial cell to leucocyte ratio <1:2.5 were considered minimally contaminated with oropharyngeal materials and were acceptable for downstream procedures.

***Measurement of 24-hour sputum volume***

Patients were instructed to collect sputum over 24 hours in sterile transparent plastic pot (50ml), which was submitted for evaluating the 24-hour sputum volume in the next out-patient visit by the attending chest physician.

**DNA extraction**

For better DNA extraction, DNA was extracted from 0.1g sputum by HiPure Fungal DNA Kit (Magen), following the modified protocol as described below. The original protocol was provided only in Chinese in <http://www.magentec.com.cn/product_show.php?id=71> . Quality and quantity of the DNA was determined by Nanodrop One UV–vis spectrophotometer (Thermo Scientific).

(1) Preparation of homogenization solution: 4% NaOH solution and 2.94% sodium citrate solution were prepared separately using sterile water and subsequently mixed in equal volumes. N-acetyl-L-cysteine (NALC) powder was added to the mixture to achieve a final NALC concentration of 0.5%. 0.5% NALC-NaOH solution was decontaminated through a 0.22 μm syringe filter (Millex) and used for subsequent sputum homogenization.

(2) Homogenisation of sputum: 0.2 ml of sputum was subject to the addition of 2-3 times the volume of NALC-NaOH (depending on the homogenization effect), followed by mixing. Add 5 times the volume of PBS to the homogenized sputum for further dilution.

(3) Collection of sputum sediment: Centrifuge the sputum at 20,000g for 30 min at 4°C using an Allegra 64R high-speed refrigerated tabletop centrifuge (Beckman Coulter, USA), followed by discarding the supernatant;

(4) Preparation of the grinding tubes: 700 mg of 0.4-0.6 mm beads, four 3 mm beads and one 5 mm cobalt oxide grinding bead (Guangzhou Luca Co., Ltd.) were placed into the grinding tubes.

(5) Resuspension and grinding of sputum sediment: Remove the Buffer STE solution from the Hipure Fungal DNA kit (Magen), aspirate 400μL and add to the sputum sediment for resuspension, followed by transferral to the grinding tube. The tube was placed into a cryomill (Luca Guangzhou) and the parameters were set as follows: grinding at 75 Hz for 30 s, stopped for 20 s to cool down, repeat for 4 times for 2 min. Following completion of centrifugation at a medium speed and transfer the liquid in the grinding tube to a new EP tube.

(6) Add 40µL of Buffer SDS to the sample, mixed and processed at 95°C for 15min;

(7) Add 700µL of Buffer PBS (diluted with anhydrous ethanol) to the sample and mix well by blowing;

(8) Position the DNA binding column in the collection tube; transfer half of the mixture into the column and centrifuge at 8,000g for 1min;

(9) Discard the filtrate and position the column into the collection tube; transfer the remaining mixture to the column and centrifuge at 8,000g for 1min;

(10) Discard the filtrate and position the column into the collection tube; centrifuge at 13,000g for 2.5min;

(11) Transfer the column to a new 1.5 mL tube; add 50 µL of Elution Buffer to the centre of the column and allow to stand for 5 min; centrifuge at 13,000g for 1 min;

(12) Transfer the filtrate to the centre of the membrane of the column and allow to stand for 5 minutes at 13,000g for 1min;

(13) Transfer the filtrate to a new EP tube (nucleic acid sample); store the nucleic acid sample at -80℃ for subsequent use.

***16S rRNA gene sequencing***

**(1) PCR amplification**

1) Fragment and Primers: To amplify the V3V4 high variant region, with a fragment size of 470bp (with barcode), of the 16S rDNA gene from bacterial DNA, PCR was used with the modified primers 338F (5’-ACTCCTACGGGGAGGCAGCA-3’) and 806R (5’-GGACTACHVGGGTWTCTAAT-3’).

2) PCR reaction system: The PCR reaction system contained 25 μL of 2× Premix Taq (Takara Biotechnology, Dalian Co., Ltd., China), 1 μL of each primer (10 μM) and 3 μL of DNA (around 50 ng) template. Each PCR reaction was run in a Biometro TONE 96G (Analytik-Jena, Germany), with the amplification mainly achieved by thermal cycling, as follows: initialization at 94 ◦C for 5 min; 30 cycles of denaturation at 94 ◦C for 30 s, annealing at 52 ◦C for 30 s and extension at 72 ◦C for 30 s; followed by a final elongation at 72 ◦C for 10 min. Three technical replicates of each sample (together with water control, named CK) were amplified this way, and their ensuing PCR products mixed on a per sample basis if there was no visible amplification from water control. Fragment lengths and concentrations of the PCR products were detected by 1.5 % agarose gel electrophoresis. Those samples with a bright primary band (470 bp) were retained for use in further experiments.

**(2) Pooling and pelleting**: GeneTools analysis software (v4.03.05.0, SynGene) was used to compare the concentrations of PCR products, to calculate the volume required per sample (according to the equal mass principle), and to mix the PCR products in equal density ratios. The E.Z.N.A. Gel Extraction Kit (Omega, USA) was then used to recover the PCR mix.

**(3) Library construction and sequencing**: Library construction was performed according to the standard procedure of NEBNext® Ultra™ IIDNA Library Prep Kit for Illumina® (New England Biolabs, USA). The library was then sequenced on the Illumina Nova 6000 platform (Guangdong Magigene Biotechnology Co., Ltd. Guangzhou, China) and a 250-bp paired read code generated. All sequence data associated with this project were deposited in GenBank (PRJNA1007275).

***Reagent controls***

As the figure shown below, a negative control (CK, lab purified water) was established during the PCR amplification (**Figure SS1**). The PCR products were sequenced only after ensured that there was no visible amplification form negative control. In addition, reagent controls for extraction (3 samples, no sputum material) and PCR amplification (3 samples, no DNA template) were included and subsequently sequenced to identify any potential contaminating bacterial species. A total of 80 ASVs were annotated to the genus level, with 15 genera exhibiting mean relative abundances exceeding 0.01 (**Figure SS2**). Except for *Pseudomonas* (mean relative abundance 0.04), no other genus had a mean relative abundance greater than 0.01 in patient samples.


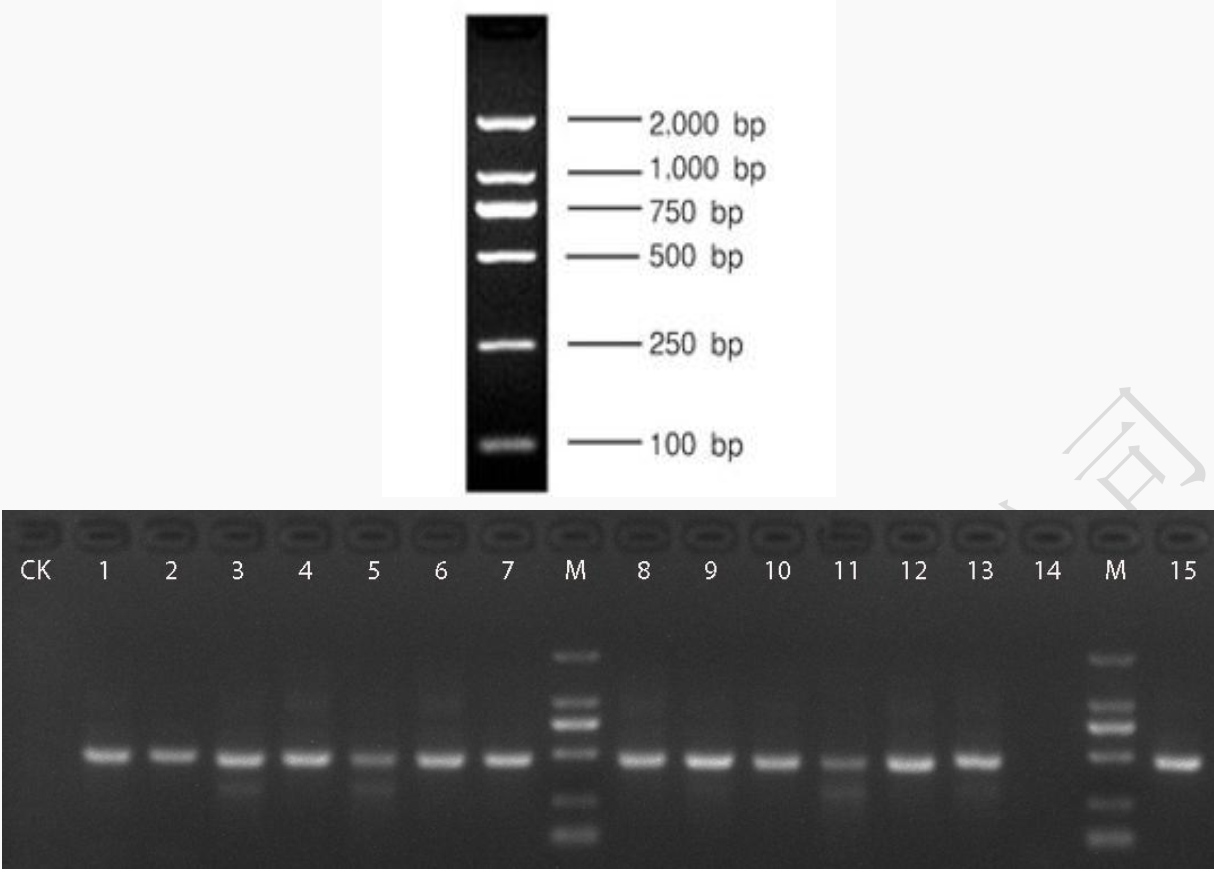


Figure A. PCR product electrophoresis graph

Note: CK denoted negative control (lab purfied water); M denoted marker reference (DL2000).


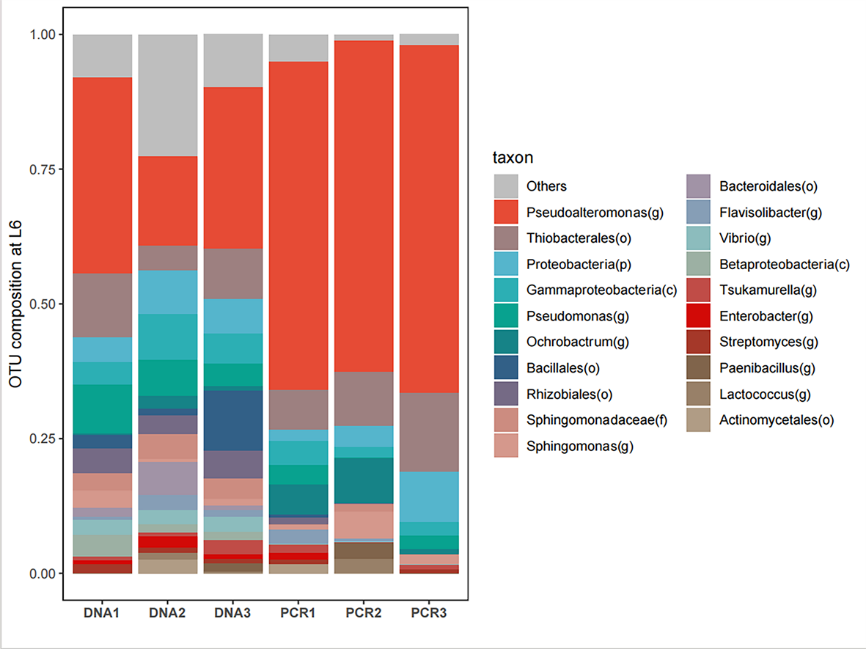


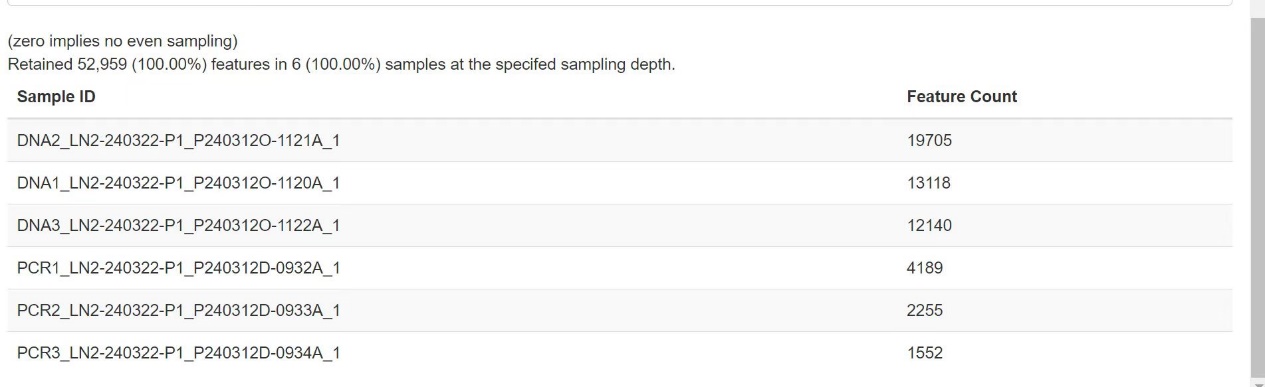


Figure B. Microbiota profiles of negative controls

***Microbiota analysis***

The 16S rRNA gene were processed by using a standardized pipeline in QIIME 2.0 (Quantitative Insights in Microbial Ecology 2.0). Demultiplexed sequencing reads were denoised to generate high-quality amplicon sequence variants (ASVs) using the Divisive Amplicon Denoising Algorithm 2 (DADA2) with default parameters. In contrast to traditional operational taxonomic unit (OTU) clustering-based methods, DADA2 used a model-based, clustering-free approach to correct for the amplicon errors and the group unique sequences into ASVs. This may identify taxonomic variations at a finer-scale. For taxonomic classification of ASVs, a custom Naïve Bayes classifier was trained on the Greengenes 13_8 99% OTUs and applied to assign the taxonomy for the ASVs in the dataset. Singletons, mitochondria and chloroplast ASVs were filtered. The QIIME2 commands used to process the data are shown below (using 250bp V4 region paired-end sequencing reads as example):

**1.Sequencing data import using prepared manifest file:**

$ qiime tools import --type 'SampleData[PairedEndSequencesWithQuality]' --input-path manifest.txt --output-path paired-end-demux.qza --input-format

PairedEndFastqManifestPhred33

**2.DADA2 denoise**, default parameter of dada2，no read length filtering was applied:

$ qiime dada2 denoise-paired --i-demultiplexed-seqs paired-end-demux.qza --p-trim-left-f 0 --p-trim-left-r 0 --p-trunc-len-f 0 --p-trunc-len-r 0 --o-table table-dada2.qza --o representative-sequences rep-seqs-dada2.qza --o-denoising-stats denoising-stats.qza --p-n-threads 10

**3.Denoise statistics visualization:**

E3$ qiime metadata tabulate --m-input-file denoising-stats.qza --o-visualization denoising_stats.qzv

**4.Extract Greengene 13_8 99% reference sequences according to primers andsequence length:**

$ qiime feature-classifier extract-reads --i-sequences 99_otus.qza --p-f-primer GTGCCAGCMGCCGCGGTAA --p-r-primer GGACTACHVGGGTWTCTAAT --p-trunc-len 250 --p-min-length 100 --p-max-length 400 --o-reads 99_otu_v4_250bp_ref_seqs.qza

**5.Train Naïve bayes classifier:**

$ qiime feature-classifier fit-classifier-naive-bayes --i-reference-reads

99_otu_v4_250bp_ref_seqs.qza --i-reference-taxonomy 99_otu_taxonomy.qza --o classifier classifier_v4_250bp.qza

**6.Apply classifier for taxonomy identification:**

$ qiime feature-classifier classify-sklearn --i-classifier classifier_v4_250bp.qza --i-reads rep-seqs-dada2.qza --o-classification taxonomy.qza

**7.Filter singletons:**

$ qiime feature-table filter-features --i-table table-dada2.qza --p-min-samples 2 --o filtered-table table-dada2-min2.qza

**8.Remove chloroplast and mitochondria sequences:**

qiime taxa filter-table --i-table table-dada2-min2.qza --i-taxonomy taxonomy.qza --p-exclude mitochondria,chloroplast --o-filtered-table table-dada2-final.qza

**9.Taxonomy collapse to species (L6) level:**

$ qiime taxa collapse --i-table table-dada2-final.qza --i-taxonomy taxonomy.qza --p-level 6 --o-collapsed-table table-dada2-final-l6.qza

**10.Rarefaction (for within datasets statistical analysis, using 32137 rarefaction depth as example):**

$ qiime feature-table rarefy --i-table table-dada2-final.qza --p-sampling-depth 32137 --o-rarefied-table table-dada2-final-rare32137.qza

**11.Export representative sequences:**

$ qiime tools export --input-path rep-seqs-dada2.qza --output-path rep-seqs-dada2

**12.Export ASV table into biom file:**

$ qiime tools export --input-path table-dada2.qza --output-path table-dada2

**13.Alpha and beta diversity analysis:**

$ qiime diversity core-metrics-phylogenetic --i-phylogeny rooted-tree.qza --i-table table-dada2-final.qza --p-sampling-depth 32137 --m-metadata-file metadata.txt --output-dir core-metrics-results

***Differential species analysis***

Rarefactions, alpha diversity (within sample evenness – Shannon diversity index), and beta diversity (differences in taxa between samples – UniFrac distance) calculations were all performed with the same QIIME pipeline. In total, we obtained 81,769,907 high-quality 16S rRNA gene reads [median: 113,118 (range: 32,137-252,716) per sample]. Samples were rarefied to 32,137 reads, which corresponded to the minimum number of aligned reads to a sample passing quality standards. 4,564 ASVs were identified and 305 ASVs which annotated to genus level were identified in the following taxonomic assignment. The Shannon diversity index and weighted/unweighted UniFrac distances were computed at rarefied level. Linear discriminant analysis (LDA) effect size (LEfSe) were used to identify genera that contributed significantly to the groupings. The LDA significance threshold was set at >2.0.

***Microbial co-occurrence network***

The co-occurrence analysis on microbial genera was performed using SparCC (Sparse Correlations for Compositional data) algorithm with a bootstrap procedure repeated for 100 times. Only the ASVs with at least 10% prevalence and average relative abundance greater than 0.01% were included in co-occurrence analysis. We utilized the SparCC3 package (<https://github.com/JCSzamosi/SparCC3>) to compute the correlations, which included 100 bootstrap resampling to assess the robustness of the correlations, and permutation testing was applied to obtain reliable p-values. Significant inter-taxa Spearman’s correlations with coefficients >0.3 (false discovery rate [FDR]-adjusted *P*<0.05) were displayed by co-occurrence network graphics using the Gephi software.

***Metabolomics data analysis***

The sputum samples, stored at -80°C freezers, were thawed on ice and vortexed for 10sec. 100μL each of the samples and extraction solution (Acetonitrile/Methanol 1:4, V/V) containing the internal standards were added into 2mL microcentrifuge tubes, followed by vortex for 3min and centrifugation at 12000 rpm for 10min at 4°C. 150μL of supernatant was placed in -20°C for 30min, and centrifuged at 12,000 rpm for 3min at 4°C. 120μL supernatant was transferred for liquid chromatography - mass spectrometry via a customized analysis system (Wuhan MetWare Biotechnology Co., Ltd., Wuhan, China). In each cycle, 12 precursor ions whose intensity >100 were selected for fragmentation at 30V.

The triple time-of-flight mass spectrometer was applied, with the acquisition software (TripleTOF 6600, AB SCIEX) that continuously evaluated the full-scan survey mass spectrometer data. In each cycle, 12 precursor ions whose intensity being greater than 100 were selected for fragmentation at the collision energy of 30V. The ESI source conditions were set as follows: Ion source gas 1 as 50Psi, Ion source gas 2 as 50 Psi, Curtain gas as 25 Psi, source temperature 500°C, Ion Spray Voltage Floating (ISVF) 5500V or -4500V in positive or negative modes, respectively.

A 120μL aliquot of supernatant was transferred for liquid chromatography-electrospray ionization tandem mass spectrometry (LC-ESI-MS/MS) via a customized analysis system (courtesy by Wuhan MetWare Biotechnology Co., Ltd., Wuhan, China), for metabolite detection and identification. Mass spectrometry data analysis was performed by Wuhan MetWare Biotechnology Co., Ltd., and all analytical data were processed by using Analyst 1.63 software (Sciex, Framingham, MA, USA) based on the self-built database MetWare (MWDB, Wuhan MetWare Biotechnology Co., Ltd.).

Metabolites were identified based on the internal databases and public databases, and metabolites results are listed in our GitHub data set. Based on a Principal Component Analysis separation-optimization model, the orthogonal partial least squares discrimination analysis containing score plots and permutation plots was generated. Variable importance in projection (VIP) values were extracted from the results of the orthogonal partial least squares discrimination analysis. Some analysis was performed using the **OmicStudio tools at** [**https://www.omicstudio.cn/tool**](https://www.omicstudio.cn/tool)**.** More details were provided in the online supplement or deposited in GitHub under <https://github.com/Dr-Hezf/CBA-microbiota.git>.

**Figure legends**

**
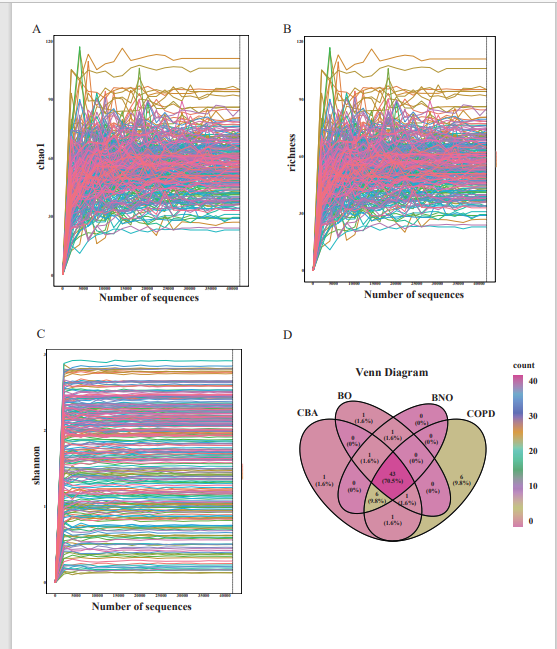
**

**Figure S1. The species accumulation (based on chao1, richness and shannon index separately) and the Venn diagram showing the overlapped bacterial genera across the four groups.**

**
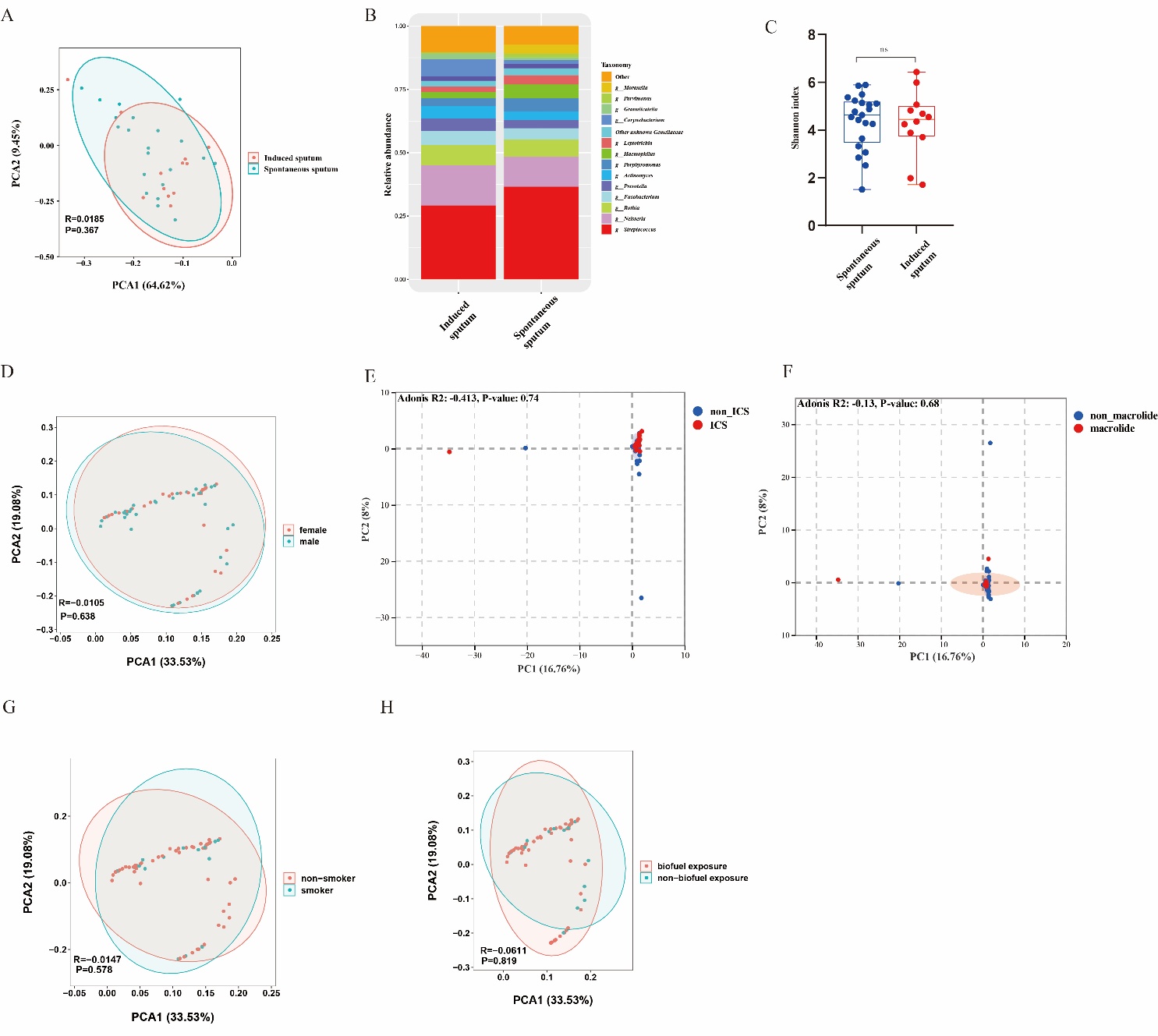
**

**Figure S2. The confounding factors of airway microbiome**.

Of 33 patients with COPD, 12 of them provided induced sputum. To assess the impact of sputum collected methods on airway microbiome, we performed principal component analysis (A), and presented the genus-level microbiome profiles (B) and alpha diversity (C) between induced and spontaneous sputum samples at stable state. Similarly, we assessed the impact of gender (D-F), smoking status (G) and biofuel-exposure (H) on the airway microbiota compositions.


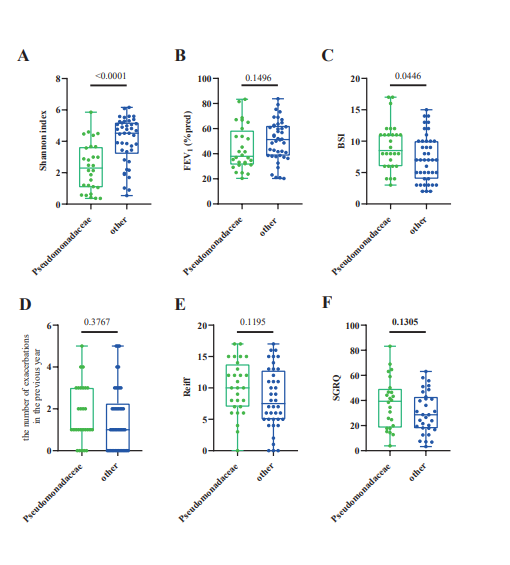


**Figure S3. Comparison of the clinical characteristics between the *Pseudomonas*-dominant and other genus-dominant CBA.**

Comparison of the Shannon-Wiener Diversity Index (SWDI, A), FEV1% predicted (B), BSI (C), Number of exacerbations in the previous year (D), Reiff score (E) and SGRQ total score (F) between the *Pseudomonas*-dominant (*Pseudomonas* being the highest OTU%, n=28) and other genus-dominant subgroups (other genera being the highest OTU%, n=42).

BSI: Bronchiectasis severity index; SGRQ: St George's Respiratory Questionnaire.


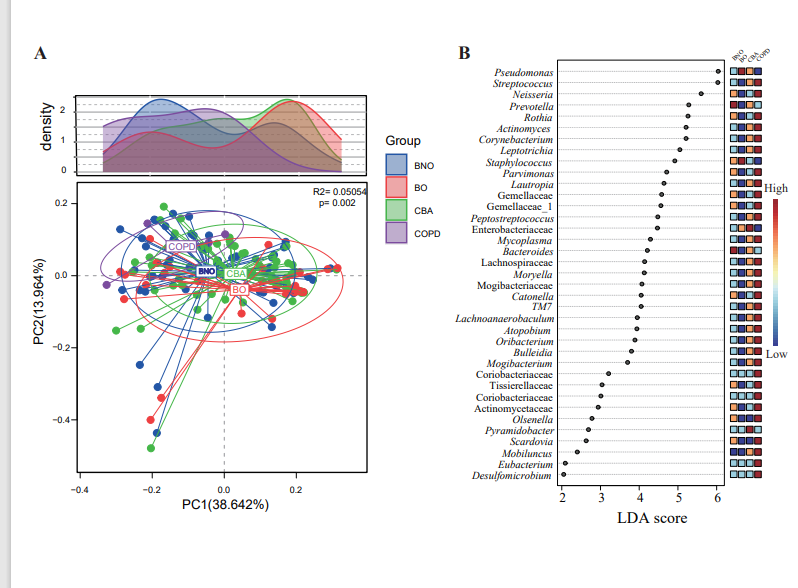


**Figure S4. Microbial profiles across four groups at exacerbation onset.**

(A) Principal coordinate analysis (PCoA) based on the weighted unifrac value for sputum at exacerbations. The microbiota significantly differed across the four groups (permutational ANOVA, R^2^=0.050, *P*=0.002). The density plot shows a more diverse first principal component (PC1) distribution for COPD than for other groups.

(B) Linear discriminant analysis (LDA) effect size (LEfSe) analysis showing the bacterial genera specifically enriched in the four groups (LDA >2.0, false discovery rate [FDR] *P*<0.05).

(C) The average relative abundance which is ranked according to the descending order is shown for each genus across the four groups.

BNO: bronchiectasis without airflow obstruction. BO: bronchiectasis with airflow obstruction. COPD: chronic obstructive pulmonary disease; CBA: COPD-bronchiectasis association.


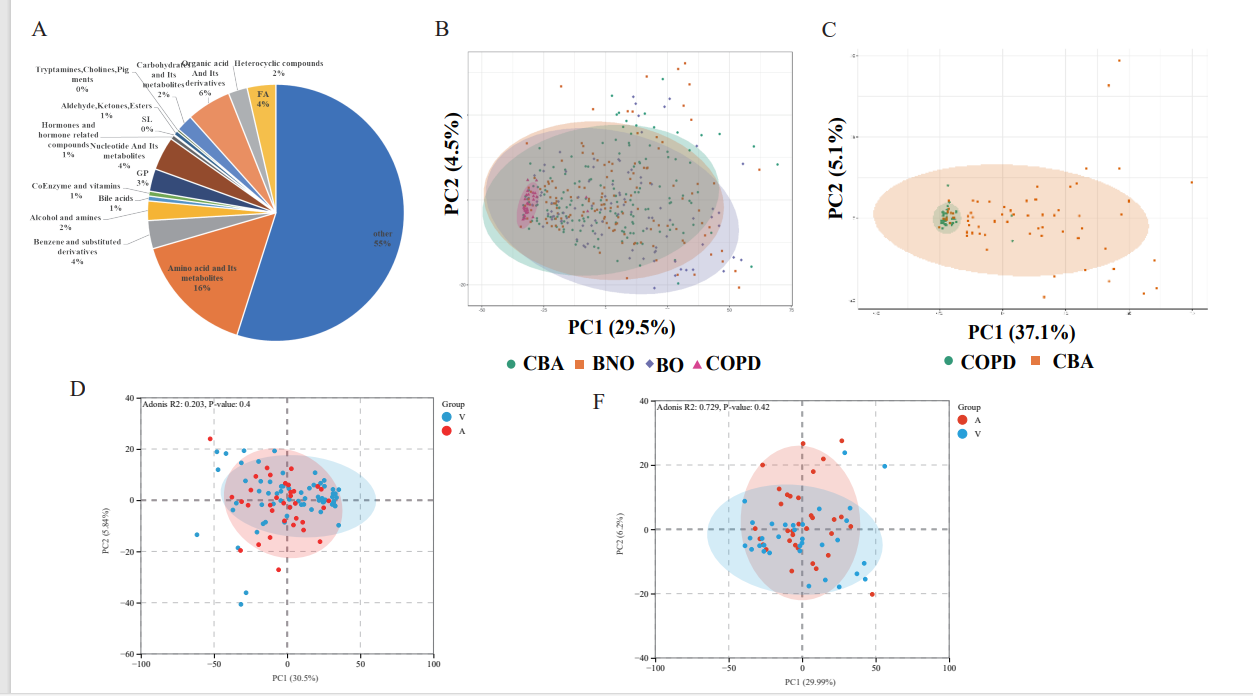


**Figure S5. Profiling of the metabolome across the four groups.**

1. Profiling of the metabolome from sputum.
2. Principal component analysis (PCA) displaying the inter-group discrimination of the four groups by the first two PCs.
3. PCA displaying the inter-group discrimination of CBA and COPD groups by the first two PCs.
4. PCA displaying the inter-group discrimination of exacerbation samples, separately with whole steady-state samples or with paired stable sample from the same subjects (E), among CBA patients by the first two PCs.
5. The number of the inter-group differentially expressed metabolites of every matched groups.

CBA: COPD-Bronchiectasis association.

**
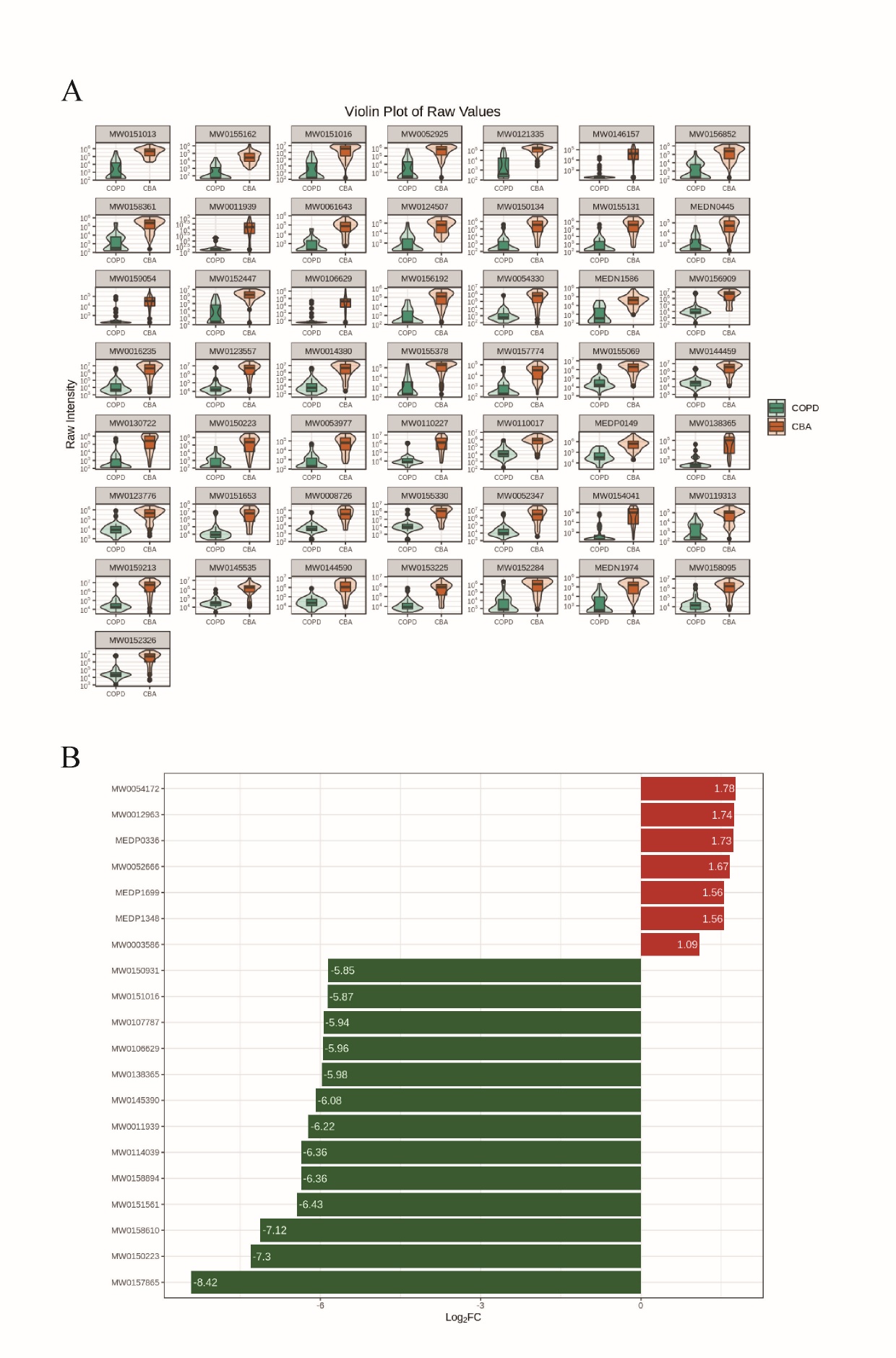
**

**Figure S6. The top 50 differential metabolites between COPD and CBA groups.**

(A) Top 50 differential metabolites with highest variable importance projection between COPD and CBA groups.

(B) Top 20 differential metabolites with highest fold-change value between COPD and CBA groups.

**
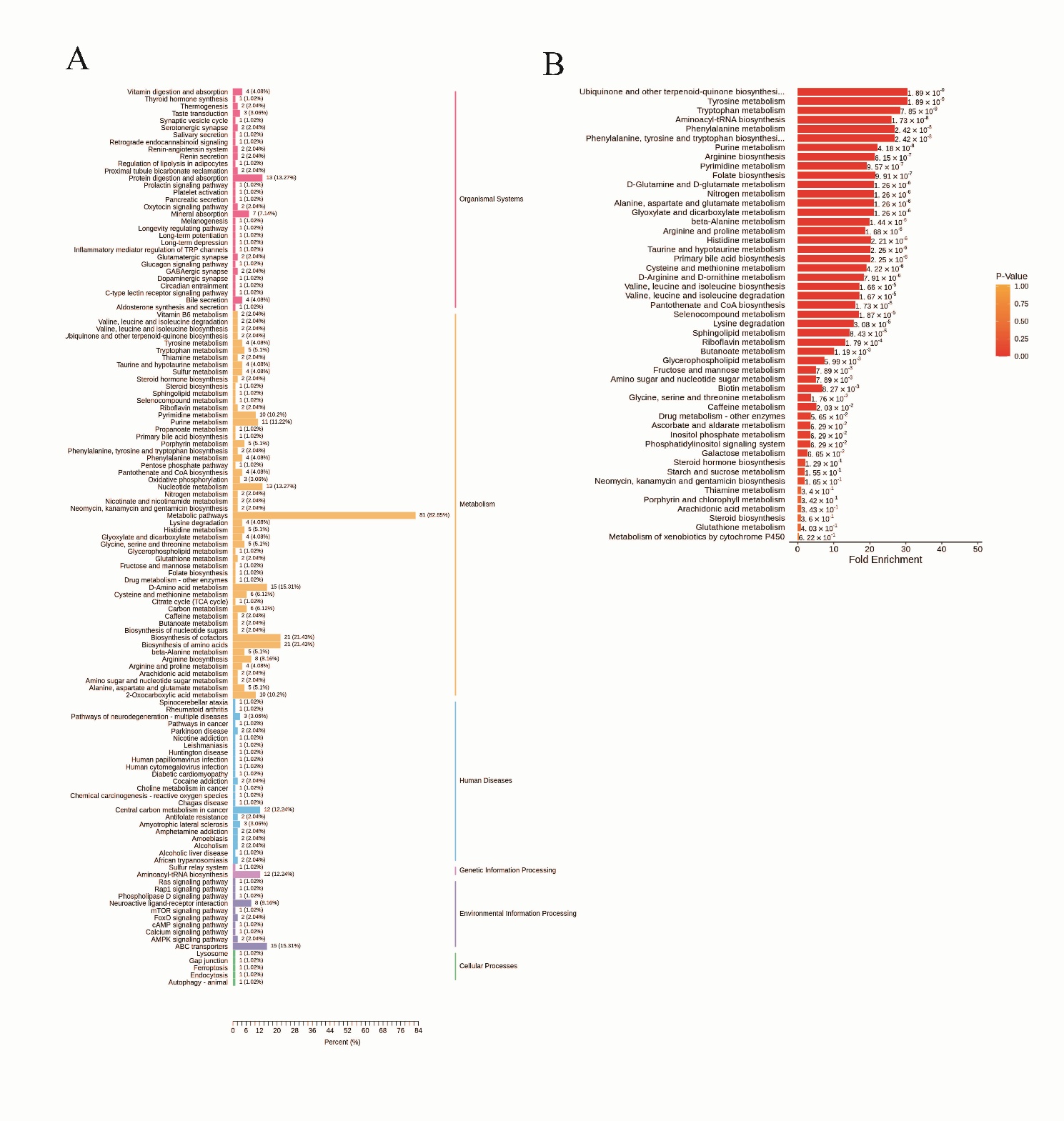
**

**Figure S7. Classification of the differential metabolites classified into the Kyoto Encyclopedia of Genes and Genomes metabolic pathways between COPD and CBA groups.**

CBA: COPD-Bronchiectasis association;


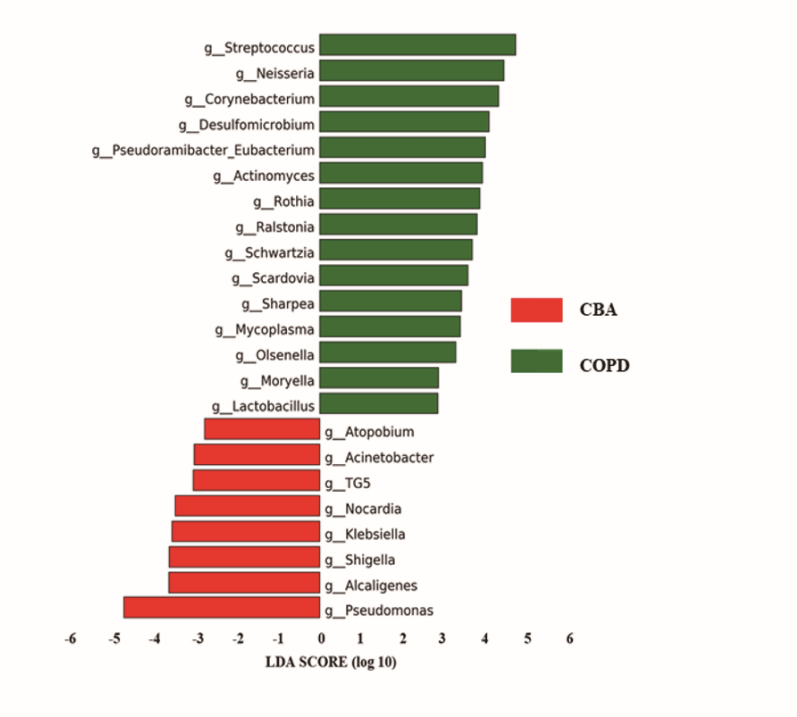


**Figure S8. LEfSe analysis identifies the differential taxa between CBA and COPD with an LDA score (Log10) of greater than 2.**

CBA: COPD-Bronchiectasis association; LEfSe: Linear discriminant analysis Effect Size

**Table S1. The *envfit* analysis in Redundancy analyses of the microbiota compositions in CBA**

| **Clinical factors** | **RDA1** | **RDA2** | **r^2^** | **P value** |
| --- | --- | --- | --- | --- |
| **BMI** | 0.76369 | 0.645584 | 0.175355 | 0.002999 |
| **disease duration** | 0.834857 | -0.55047 | 0.135397 | 0.009995 |
| **number of infected lobe** | 0.140582 | -0.99007 | 0.09641 | 0.034983 |
| **FEV_1_/FVC** | 0.455012 | -0.89049 | 0.085524 | 0.052474 |
| **FVC%pred** | -0.80755 | 0.589804 | 0.081105 | 0.057971 |
| **exacerbation frequency** | 0.96479 | -0.26302 | 0.072633 | 0.087456 |
| **Reiff** | 0.220348 | -0.97542 | 0.045427 | 0.2004 |
| **24h-sputume volume** | 0.997881 | 0.065072 | 0.039324 | 0.255372 |
| **blood eosinophil count** | 0.752017 | 0.659144 | 0.020946 | 0.514743 |
| **FEV_1_%pred** | -0.94781 | 0.318832 | 0.01865 | 0.54073 |
| **MMEF%pred** | 0.995463 | -0.09515 | 0.005216 | 0.831584 |
| **SGRQ** | 0.137821 | 0.990457 | 0.002847 | 0.916042 |
| **blood neutrophil count** | 0.335021 | -0.94221 | 0.001807 | 0.941029 |

Definition of abbreviations: CBA=COPD-Bronchiectasis association; BMI = body mass index; FEV1=The forced expiratory volume in a 1-second; FVC= forced vital capacity; SGRQ=St George's Respiratory Questionnaire.

**Table S2. Demographic and clinical characteristics of** ***Pseudomonas*-dominant and non-*Pseudomonas*-dominant CBA**

| **Parameters** | ***Pseudomonas*-dominant (N=28)** | **Non-*Pseudomonas*-dominant (N=42)** | ***P* value^$^ (CBA vs COPD)** |
| --- | --- | --- | --- |
| **Age (yr)** | 55±11 | 53±12 | 0.49 |
| **BMI (kg/m^2^)** | 21.5±3.6 | 21.3±2.8 | 0.81 |
| **Gender (% female)** | 14(50%) | 25 (60%) | ns |
| **Smoking status^†^**  Never-smokers (No., %)  Ex- or current-smokers (No., %) | 22(78.6%)  6(21.4%) | 31(73.8%)  11(26.2%) | ns |
| **Exposure of biomass fuel^††^** | 12(42.9%) | 26(61.9%) | ns |
| **FEV_1_% predicted** | 44.5±17.7 | 50.5±16.2 | 0.15 |
| ***PA* colorization, n (%) ^‡‡^** | 22(78.6%) | 6(14.3%) | ** |
| **Exacerbation frequency in the past year** | 1(0-3) | 1(0-3) | 0.37 |
| **CAT score** | 13.2±6.7 | 12.1±7.3 | 0.59 |
| **SGRQ score** | 37.8±20.4 | 30.2±16.7 | 0.13 |
| **Bronchiectasis severity index** | 9.18±3.78 | 7.31±3.73 | 0.045 |
| **HRCT Reiff score** | 10.1±4.3 | 8.3±4.8 | 0.12 |

Definition of abbreviations: BNO= Bronchiectasis without airflow obstruction; BO= Bronchiectasis with airflow obstruction; CBA=COPD-Bronchiectasis association; COPD= Chronic obstructive pulmonary disease; BMI = body mass index; FEV_1_=The forced expiratory volume in a 1-second; FVC= forced vital capacity; *PA*= *Pseudomonas aeruginosa*; CAT = COPD Assessment Test; SGRQ=St George's Respiratory Questionnaire; HRCT=High Resolution computerized tomography.

**^†^** Patient demographic data at baseline, ex- or current-smoker had at least 10 pack-years;

**^††^** Patient demographic data at baseline, defined as with biomass exposure for at least 10 years.

**^‡^** Patient demographic data at baseline, defined as the time from the first respiratory symptom onset.

**^‡‡^** Defined as the appearance of positive culture of *PA* at least 2 occasions at least 3 months apart within 1 year

**^$^** Multiple comparisons were performed with the Bonferroni correction for homoscedastic data and Tamhane correction for heteroscedastic data.

**Table S3. Analysis of future exacerbation risk using Kaplan-Meier curve**

| **Reference** | **Tested group** | **Hazard ratio (95%CI), P value** |
| --- | --- | --- |
| **CBA-other** | CBA-PA | 2.461 (1.341-4.516), P<0.001 |
| **BO-other** | BO-PA | 2.005 (1.054-3.816), P=0.034 |
| **BO-PA** | CBA-PA | 1.818 (1.002-3.298), P=0.049 |
| **BO-other** | CBA-other | 1.482 (0.820-2.677), P=0.207 |

Definition of abbreviations: BNO: bronchiectasis without airflow obstruction; BO=Bronchiectasis with airflow obstruction; CBA=COPD-Bronchiectasis association; COPD: chronic obstructive pulmonary disease. *P<0.05, **P<0.01, ***P<0.001

**Table S4. Results of the Cox proportional hazards regression analysis for exacerbation in CBA and BO patients**

| **Variable** | **CBA** | | |  | **BO** | | |
| --- | --- | --- | --- | --- | --- | --- | --- |
|  | **Hazard Ratio (HR)** | **95% Confidence Interval (CI)** | ***P*-value** |  | **Hazard Ratio (HR)** | **95% Confidence Interval (CI)** | ***P*-value** |
| Pseudomonadaceae_dominant | 2.295 | 1.2426 - 4.240 | 0.0080 ** |  | 2.708 | 1.191 - 6.159 | 0.018 * |
| BSI | 1.047 | 0.958 - 1.144 | 0.306 |  | 1.214 | 1.035 - 1.425 | 0.018 * |
| CAT | 0.011 | 0.972 - 1.053 | 0.567 |  | 0.983 | 0.914 - 1.058 | 0.647 |
| Age | 0.990 | 0.958 - 1.023 | 0.548 |  | 0.945 | 0.898 - 0.996 | 0.033 * |
| BMI | 0.981 | 0.884 - 1.090 | 0.731 |  | 1.242 | 1.019 - 1.514 | 0.032 * |
| FEV_1_ | 0.954 | 0.296 - 3.075 | 0.938 |  | 0.870 | 0.080 - 9.432 | 0.909 |
| FVC | 1.087 | 0.434 - 2.721 | 0.858 |  | 0.976 | 0.222 - 4.285 | 0.974 |
| Reiff score | 1.059 | 0.990 - 1.133 | 0.093 |  | 0.933 | 0.833 -1.046 | 0.235 |

Definition of abbreviations: BSI= Bronchiectasis Severity Index; BMI = body mass index; FEV_1_=The forced expiratory volume in a 1-second; FVC= forced vital capacity; CAT = COPD Assessment Test; BO=Bronchiectasis with airflow obstruction; CBA=COPD-Bronchiectasis association; **P*<0.05, ***P*<0.01, ****P*<0.001

**Table S5. Top 50 differential metabolites with the highest variable importance projection values between COPD and CBA.**

| **Index** | **Compounds** | **Class I** | **VIP** | **p_value** | **FDR** | **Fold_Change** | **Log2FC** | **Type** |
| --- | --- | --- | --- | --- | --- | --- | --- | --- |
| MW0151013 | His-Lys-Ile-Asp | Amino acid and Its metabolites | 1.686 | 2.17E-13 | 1.95E-10 | 0.047 | -4.412 | down |
| MW0155162 | Phe-Gln-Ile | Amino acid and Its metabolites | 1.644 | 6.44E-13 | 2.9E-10 | 0.019 | -5.736 | down |
| MW0151016 | His-Lys-Met | Amino acid and Its metabolites | 1.631 | 2.83E-12 | 4.74E-10 | 0.017 | -5.865 | down |
| MW0052925 | Fluorometholone | Aldehyde,Ketones,Esters | 1.612 | 7.36E-12 | 5.83E-10 | 0.062 | -4.000 | down |
| MW0121335 | 5-Chloro-1-(piperidin-4-yl)-1H-benzo[d]imidazol-2(3H)-one | Benzene and substituted derivatives | 1.607 | 3.16E-12 | 4.74E-10 | 0.113 | -3.140 | down |
| MW0146157 | Asp-Ile-Ser-Glu | Amino acid and Its metabolites | 1.601 | 1.27E-12 | 4.55E-10 | 0.018 | -5.776 | down |
| MW0156852 | Ser-Lys-Tyr | Amino acid and Its metabolites | 1.586 | 4.16E-12 | 5.34E-10 | 0.036 | -4.812 | down |
| MW0158361 | Tyr-Ala-Phe-Arg | Amino acid and Its metabolites | 1.582 | 6.61E-12 | 5.83E-10 | 0.049 | 4.360 | down |
| MW0011939 | Estradiol-17beta-glucuronide | Aldehyde,Ketones,Esters | 1.580 | 1.72E-13 | 1.95E-10 | 0.013 | -6.222 | down |
| MW0061643 | Physalolactone C | Others | 1.580 | 2.21E-12 | 4.67E-10 | 0.024 | -5.388 | down |
| MW0124507 | Roquefortine C | Heterocyclic compounds | 1.578 | 3.73E-12 | 5.16E-10 | 0.058 | -4.098 | down |
| MW0150134 | Glu-Ser-Arg-Asp | Amino acid and Its metabolites | 1.568 | 2.33E-12 | 4.67E-10 | 0.022 | -5.494 | down |
| MW0155131 | Phe-Asp-Ile-Glu | Amino acid and Its metabolites | 1.568 | 2.33E-12 | 4.67E-10 | 0.022 | -5.494 | down |
| MEDN0445 | Orotidine | Nucleotide And Its metabolites | 1.558 | 1.19E-11 | 6.51E-10 | 0.040 | -4.651 | down |
| MW0159054 | Val-Leu-Ala-Leu-Leu | Amino acid and Its metabolites | 1.557 | 1.13E-11 | 6.37E-10 | 0.088 | -3.505 | down |
| MW0152447 | Leu-Phe-Glu | Amino acid and Its metabolites | 1.545 | 1.78E-11 | 7.09E-10 | 0.060 | -4.065 | down |
| MW0106629 | HMB-Val-Ser-Leu-VE | Benzene and substituted derivatives | 1.544 | 2.34E-12 | 4.67E-10 | 0.016 | -5.955 | down |
| MW0156192 | PtdIns-(1,2-dioctanoyl)(sodium salt) | Organic acid And Its derivatives | 1.538 | 8.42E-12 | 5.83E-10 | 0.024 | -5.386 | down |
| MW0054330 | 5S-Hydroxy-6R-(S-gamma-glutamylcysteinyl)-7E,9E,11Z,14Z-eicosatetraenoic acid | FA | 1.533 | 7.56E-12 | 5.83E-10 | 0.026 | -5.287 | down |
| MEDN1586 | Thymidine-5'-phosphate (dTMP) | Nucleotide And Its metabolites | 1.533 | 2.52E-11 | 7.89E-10 | 0.058 | -4.105 | down |
| MW0156909 | Ser-Ser-Gly-Arg-Ser | Amino acid and Its metabolites | 1.524 | 1.13E-11 | 6.37E-10 | 0.059 | -4.093 | down |
| MW0016235 | Boviquinone 4 | Aldehyde,Ketones,Esters | 1.523 | 1.93E-11 | 7.09E-10 | 0.028 | -5.176 | down |
| MW0123557 | DESACETYL(7)KHIVORINIC ACID,METHYL ESTER | Organic acid And Its derivatives | 1.521 | 4.59E-11 | 8.24E-10 | 0.058 | -4.106 | down |
| MW0014380 | 4-{[(4S,5R,6E,8E,10Z,13Z)-1-carboxy-4-hydroxynonadeca-6,8,10,13-tetraen-5-yl]sulfanyl}benzoic acid | CoEnzyme and vitamins | 1.521 | 1.74E-11 | 7.09E-10 | 0.028 | -5.153 | down |
| MW0155378 | Phe-Tyr-Asn-Glu | Amino acid and Its metabolites | 1.521 | 2.2E-11 | 7.47E-10 | 0.077 | -3.696 | down |
| MW0157774 | Thr-Phe-Val-Arg | Amino acid and Its metabolites | 1.517 | 1.48E-11 | 7.01E-10 | 0.070 | -3.835 | down |
| MW0155069 | Phe-Ala-Arg-Asp | Amino acid and Its metabolites | 1.514 | 1.4E-11 | 7.01E-10 | 0.067 | -3.905 | down |
| MW0144459 | Ala-Ala-Lys-Ser-Asp | Amino acid and Its metabolites | 1.514 | 1.44E-11 | 7.01E-10 | 0.037 | -4.768 | down |
| MW0130722 | 2-benzyl-4-(octahydro-1H-isoindol-2-yl)-4-oxobutanoic acid | Organic acid And Its derivatives | 1.513 | 6.09E-12 | 5.83E-10 | 0.040 | -4.635 | down |
| MW0150223 | Glu-Val-Tyr-Asp | Amino acid and Its metabolites | 1.513 | 9.13E-12 | 6.08E-10 | 0.006 | -7.300 | down |
| MW0053977 | Hydrocortisone hemisuccinate | Hormones and hormone related compounds | 1.512 | 6.6E-12 | 5.83E-10 | 0.029 | -5.127 | down |
| MW0110227 | Tyr-Tyr-Lys | Amino acid and Its metabolites | 1.510 | 4.77E-12 | 5.72E-10 | 0.027 | -5.187 | down |
| MW0110017 | TRIBUTYL PHOSPHATE | Aldehyde,Ketones,Esters | 1.502 | 1.13E-11 | 6.37E-10 | 0.042 | -4.576 | down |
| MEDP0149 | 2'-Deoxyadenosine-5'-Monophosphate | Nucleotide And Its metabolites | 1.501 | 6.26E-12 | 5.83E-10 | 0.086 | -3.544 | down |
| MW0138365 | Hernandezine | Heterocyclic compounds | 1.500 | 5.92E-12 | 5.83E-10 | 0.016 | -5.976 | down |
| MW0123776 | Dulciol A | Heterocyclic compounds | 1.499 | 2.95E-11 | 7.89E-10 | 0.067 | -3.891 | down |
| MW0151653 | Ile-Val-Lys-Trp-Asp | Amino acid and Its metabolites | 1.497 | 1.56E-11 | 7.09E-10 | 0.038 | -4.713 | down |
| MW0008726 | N-[4-[[6-Methoxy-7-[3-(4-morpholinyl)propoxy]-4-quinazolinyl]amino]phenyl]benzamide | Benzene and substituted derivatives | 1.496 | 7.37E-12 | 5.83E-10 | 0.028 | -5.144 | down |
| MW0155330 | Phe-Ser-His-Arg | Amino acid and Its metabolites | 1.495 | 4.03E-11 | 7.89E-10 | 0.046 | -4.451 | down |
| MW0052347 | Diflorasone diacetate | Aldehyde,Ketones,Esters | 1.493 | 2.95E-11 | 7.89E-10 | 0.028 | -5.175 | down |
| MW0154041 | Myxochromide S2 | Alcohol and amines | 1.488 | 1.04E-11 | 6.37E-10 | 0.036 | -4.783 | down |
| MW0119313 | 3-[2-(1-methylpyridin-1-ium-4-yl)ethenyl]-1H-indole;iodide | Heterocyclic compounds | 1.488 | 1.32E-10 | 1.04E-09 | 0.059 | -4.079 | down |
| MW0159213 | Val-Tyr-Gln-Lys | Amino acid and Its metabolites | 1.486 | 9.71E-11 | 9.45E-10 | 0.063 | -3.986 | down |
| MW0145535 | Arg-Tyr-Ala-Arg | Amino acid and Its metabolites | 1.486 | 2.79E-11 | 7.89E-10 | 0.033 | -4.914 | down |
| MW0144590 | Ala-His-Gly-Val-Asp | Amino acid and Its metabolites | 1.486 | 1.74E-11 | 7.09E-10 | 0.028 | -5.177 | down |
| MW0153225 | Lys-HoPhe-OH | Amino acid and Its metabolites | 1.486 | 2.04E-11 | 7.18E-10 | 0.043 | -4.547 | down |
| MW0152284 | Leu-Gln-Asn-Arg | Amino acid and Its metabolites | 1.486 | 3.45E-11 | 7.89E-10 | 0.057 | -4.121 | down |
| MEDN1974 | Ile-Gly | Amino acid and Its metabolites | 1.484 | 6.77E-11 | 8.53E-10 | 0.070 | -3.830 | down |
| MW0158095 | Trp-Arg-Gln | Amino acid and Its metabolites | 1.483 | 5.8E-11 | 8.49E-10 | 0.055 | -4.173 | down |
| MW0152326 | Leu-Glu-Phe-Glu | Amino acid and Its metabolites | 1.481 | 8.33E-11 | 9.12E-10 | 0.063 | -3.999 | down |

**Table S6. Top 20 differential metabolites with the highest fold-change between COPD and CBA.**

| **Index** | **Compounds** | **Class I** | **VIP** | **p_value** | **FDR** | **Fold_Change** | **Log2FC** | **Type** |
| --- | --- | --- | --- | --- | --- | --- | --- | --- |
| MW0151016 | His-Lys-Met | Amino acid and Its metabolites | 1.631 | 2.83E-12 | 4.74E-10 | 0.017 | -5.865 | down |
| MW0011939 | Estradiol-17beta-glucuronide | Aldehyde,Ketones,Esters | 1.580 | 1.72E-13 | 1.95E-10 | 0.013 | -6.222 | down |
| MW0106629 | HMB-Val-Ser-Leu-VE | Benzene and substituted derivatives | 1.544 | 2.34E-12 | 4.67E-10 | 0.016 | -5.955 | down |
| MW0150223 | Glu-Val-Tyr-Asp | Amino acid and Its metabolites | 1.513 | 9.13E-12 | 6.08E-10 | 0.006 | -7.300 | down |
| MW0138365 | Hernandezine | Heterocyclic compounds | 1.500 | 5.92E-12 | 5.83E-10 | 0.016 | -5.976 | down |
| MW0150931 | His-Glu-Tyr-Lys | Amino acid and Its metabolites | 1.476 | 1.48E-11 | 7.01E-10 | 0.017 | -5.849 | down |
| MW0145390 | Arg-Ile-Glu-Asp | Amino acid and Its metabolites | 1.464 | 1.74E-11 | 7.09E-10 | 0.015 | -6.084 | down |
| MW0158610 | Tyr-Ser-Phe-Val-Phe | Amino acid and Its metabolites | 1.439 | 3.16E-12 | 4.74E-10 | 0.007 | -7.122 | down |
| MW0157865 | Thr-Val-Leu-Thr-Ser | Amino acid and Its metabolites | 1.421 | 5.58E-13 | 2.9E-10 | 0.003 | -8.416 | down |
| MW0114039 | Carbomycin | Heterocyclic compounds | 1.385 | 5.37E-11 | 8.49E-10 | 0.012 | -6.358 | down |
| MW0158894 | Val-Arg-Glu-Glu | Amino acid and Its metabolites | 1.378 | 7.13E-11 | 8.68E-10 | 0.012 | -6.363 | down |
| MW0151561 | Ile-Pro-Phe | Amino acid and Its metabolites | 1.272 | 2.08E-09 | 6.11E-09 | 0.012 | -6.432 | down |
| MW0107787 | Leucine enkephalin acetate salt | Amino acid and Its metabolites | 1.270 | 3.41E-09 | 9.24E-09 | 0.016 | -5.942 | down |
| MW0054172 | (4E,9E)-9-(chloromethylidene)-N-[(E)-3-methoxy-5-[(2S)-2-methyl-5-oxo-2H-pyrrol-1-yl]-5-oxopent-3-enyl]-6-methyltetradeca-4,13-dienamide | FA | 1.161 | 6.25E-08 | 1.34E-07 | 3.429 | 1.778 | up |
| MEDP0336 | LPC(0:0/14:0) | GP | 1.130 | 8.82E-08 | 1.86E-07 | 3.318 | 1.730 | up |
| MW0052666 | Estrone glucuronide | Aldehyde,Ketones,Esters | 1.112 | 1.32E-07 | 2.73E-07 | 3.177 | 1.668 | up |
| MW0012963 | 1-Palmitoyl-sn-glycero-3-phosphoethanolamine | GP | 1.058 | 7.09E-07 | 1.41E-06 | 3.332 | 1.736 | up |
| MW0003586 | 3,4,5-Trimethoxyphenol | Benzene and substituted derivatives | 1.027 | 3E-05 | 5.49E-05 | 2.128 | 1.089 | up |
| MEDP1699 | LPC(0:0/16:1) | GP | 1.023 | 1.16E-06 | 2.28E-06 | 2.943 | 1.557 | up |
| MEDP1348 | LPC(16:1/0:0) | GP | 1.023 | 1.16E-06 | 2.28E-06 | 2.943 | 1.557 | up |
